# Metadherin enhances vulnerability of cancer cells to ferroptosis

**DOI:** 10.1038/s41419-019-1897-2

**Published:** 2019-09-17

**Authors:** Jianling Bi, Shujie Yang, Long Li, Qun Dai, Nicholas Borcherding, Brett A. Wagner, Garry R. Buettner, Douglas R. Spitz, Kimberly K. Leslie, Jun Zhang, Xiangbing Meng

**Affiliations:** 1 Department of Obstetrics and Gynecology, Iowa City, IA 52242 USA; 2 Department of Pathology, Iowa City, IA 52242 USA; 3 Holden Comprehensive Cancer Center, Iowa City, IA 52242 USA; 4 Department of Internal Medicine, Division of Hematology, Oncology and Blood & Marrow Transplantation, Iowa City, IA 52242 USA; 5 Division of Medical Oncology, Department of Internal Medicine, University of Kansas Cancer Center, University of Kansas Medical Center, 2330 Shawnee Mission Pkwy #210, Westwood, KS 66205 USA; 6 Medical Science Training Program (MSTP), Iowa City, IA 52242 USA; 7 Free Radical Radiation Biology, and Division of the Department of Radiation Oncology, University of Iowa Carver College of Medicine, Iowa City, IA 52242 USA; 8 Department of Cancer Biology, University of Kansas Cancer Center, University of Kansas Medical Center, 3005B Wahl Hall East, 3901 Rainbow Blvd, Kansas City, KS 66160 USA

**Keywords:** Tumour biomarkers, Cell death

## Abstract

Ferroptosis is an iron-dependent, non-apoptotic form of regulated cell death driven by lipid hydroperoxides within biological membranes. Although therapy-resistant mesenchymal-high cancers are particularly vulnerable to ferroptosis inducers, especially phospholipid glutathione peroxidase 4 (GPx4) inhibitors, the underlying mechanism is yet to be deciphered. As such, the full application of GPx4 inhibitors in cancer therapy remains challenging. Here we demonstrate that metadherin (MTDH) confers a therapy-resistant mesenchymal-high cell state and enhanced sensitivity to inducers of ferroptosis. Mechanistically, MTDH inhibited GPx4, as well as the solute carrier family 3 member 2 (SLC3A2, a system X_c_^−^ heterodimerization partner), at both the messenger RNA and protein levels. Our metabolomic studies demonstrated that MTDH reduced intracellular cysteine, but increased glutamate levels, ultimately decreasing levels of glutathione and setting the stage for increased vulnerability to ferroptosis. Finally, we observed an enhanced antitumor effect when we combined various ferroptosis inducers both in vitro and in vivo; the level of MTDH correlated with the ferroptotic effect. We have demonstrated for the first time that MTDH enhances the vulnerability of cancer cells to ferroptosis and may serve as a therapeutic biomarker for future ferroptosis-centered cancer therapy.

## Introduction

Ferroptosis is a non-apoptotic form of regulated cell death driven by lipid peroxidation, which requires abundant and accessible cellular iron^1^. As a cellular process, ferroptosis acts in pivotal roles in both normal development and various diseases, including cancer^1^. The removal of phospholipid hydroperoxides relies largely on phospholipid glutathione (GSH) peroxidase 4 (GPx4), a GSH-dependent enzyme. Importantly, GPx4 is the only known enzyme that reduces lipid hydroperoxides within biological membranes^2^, converting polyunsaturated phospholipid lipid hydroperoxide into corresponding lipid alcohol^3^. Therefore, GPx4 is considered as a central regulator of ferroptotic cell death^3^. In order to enable the function of GPx4, the production and regulation of the metabolite GSH is necessary. GSH is synthesized from cysteine and glutamate, whose intracellular concentration is fine-tuned by system x_c_^−^, an amino acid antiporter that mediates the exchange of extracellular cystine and intracellular glutamate across the cellular plasma membrane^3^. Cystine is reduced to cysteine in order to act as a substrate to synthesize GSH by glutamate cysteine ligase. System x_c_^−^ is a disulfide-linked heterodimer composed of solute carrier family 7 member 11 (SLC7A11) and SLC3A2^4^.

Ferroptosis can be induced through various modalities such as system x_c_^−^ inhibitors, compounds that deplete GSH^1^, and inhibitors of GPx4, along with other less reported mechanisms^3,5,6^. The inhibition or rescue from ferroptosis can be induced by iron chelators, reducing agents, and inhibitors of lipid peroxidation^7^. Accumulating evidence suggests that resistant, mesenchymal-high cancers are particularly vulnerable to ferroptosis inducers, especially GPx4 inhibitors^5,6^. However, the underlying mechanism is yet to be deciphered, and the full application of GPx4 inhibitors as cancer therapy remains unrealized.

Metadherin (MTDH), also known as astrocyte elevated gene-1 protein (AEG-1), is located on chromosome 8q22, a region frequently amplified in breast and hepatocellular cancers^8,9^. *MTDH* overexpression was documented in many types of cancer and correlates clinically to poor overall survival^10–12^. Several studies have established a bona fide role of MTDH in several hallmarks of cancer, including transformation, proliferation, evasion of apoptosis, and therapeutic resistance^13,14^. *MTDH* overexpression causes broad drug resistance to 5-fluorouracil, doxorubicin, cisplatin, mitomycin C, paclitaxel, histone deacetylase inhibitors, and other agents^13,15–17^, as well as resistance to radiation therapy^18^.

These diverse findings of resistance associated with *MTDH* expression underscores the pleotropic interactions of MTDH with other signaling modules and networks. MTDH may act in the nucleus as a transcription co-factor, for example, MTDH can physically interact with p65 and therefore activate nuclear factor-κB^19^. MTDH can also interact with CREB-binding protein, preventing its ubiquitin-mediated degradation and thereby facilitating the epigenetic activation of Twist-related protein 1 (TWIST1)^20^. In addition, MTDH can function as an effector of multiple epithelial–mesenchymal transition (EMT)-related microRNAs, and incorporate oncogenic signaling pathways such as phosphoinositide 3-kinase-AKT and Wnt/β-catenin to promote EMT, cancer stemness, and metastasis^12,21–24^. While conferring resistance to chemotherapy agents and radiotherapy, MTDH was found to promote the EMT, invasion, and metastasis in various types of cancers including breast cancer^22,25–27^.

As MTDH promotes a therapy-resistant, mesenchymal-high cell state, we therefore focused on whether MTDH enhances the vulnerability of cancer cells to ferroptosis inducers and the mechanism of the underlying vulnerability. This study provides evidence to support ferroptosis induction by GPx4 inhibitors can overcome MTDH-overexpression-mediated drug resistance to conventional chemotherapy and radiation therapy^13,15–18^.

## Materials and methods

### Bioinformatics using TCGA, GSEA, and CTRP

The Cancer Genome Atlas (TCGA) RNA-sequencing level 3 processed data were downloaded from UCSC Xena Browser [https://xenabrowser.net]. The messenger RNA (mRNA) expression data were sorted per MTDH expression and samples were split into tertiles. The high and low MTDH tertiles were subjected to Gene Set Enrichment Analysis (GSEA) using the C2, C5, and Hallmark libraries examining EMT and metastasis gene sets^28,29^. A false discovery rate (FDR) cutoff of <0.25 was considered to be significant. In order to correlate the mesenchymal score with the sensitivity to ferroptosis inducers, we used previously published mesenchymal scores^6^, and drug sensitivity data from the Cancer Therapeutics Response Portal (CTRP) [http://portals.broadinstitute.org/ctrp.v2.2/]. Data and visualizations utilized R (v.3.5.1) with the base functions and ggplots2 package (v.3.1.0) were used to visualize results.

### Drugs

For in vitro experiments, sorafenib (#S7397, Selleckchem, Houston, TX, USA), erastin (#5449, Tocris, Bristol, UK), sulfasalazine (SAS, #599-79-1, Cayman Chemical, Ann Arbor, MI, USA), M162 (#1035072-16-2, Cayman Chemical), M210 (#1360705-96-9, Cayman Chemical), and ferrostatin-1 (Fer-1) (#5180, Tocris) were prepared in dimethyl sulfoxide (DMSO). For in vivo studies, sorafenib was dissolved in Cremophor EL/95% ethanol (50:50, Sigma Chemical Company, St. Louis, MO, USA). The 1*S*, 3*R*-RSL3 (#6118, Tocris) and ML162 were dissolved in DMSO.

### Culture of cell lines and assessment of cytotoxicity to various ferroptosis inducers and their combinations

Non-small-cell lung carcinoma (NSCLC) cell lines A549 and H1975; small-cell lung cancer (SCLC) cell lines DMS53 and DMS273; endometrial cancer cell lines KLE, AN3CA, RL95, Hec1A, and Ishikawa; and breast cancer cell lines MDA-MB-231 and MCF-7 were purchased from the American Type Culture Collection. Hec50 uterine serous carcinoma cells were kindly provided by Dr. Erlio Gurpide in 1991 (New York University), and their identities were verified by genotyping service at Bio-Synthesis—CRO/CMO Life Sciences Company (Lewisville, TX, USA). The CRISPR approach was used to generate isogenic *MTDH* knockout (KO) cells of Hec50 and MDA-MB-231 as described previously^30^. The single guide RNA (sgRNA) CAAAACAGTTCACGCCATGA targeted the coding region of the *MTDH* gene at 97,686,713 to 97,686,733 (Sequence ID: NC_000008.11 at *Homo sapiens* chromosome 8, GRCh38.p12). The sgRNA was cloned into lentiCRISPRv1 (Addgene Plasmid 49535, Addgene, Watertown, MA, USA). The viral vectors were produced in HEK293T cells following the manufacturer’s protocol. Cells were infected with the lentivirus and cultured in the presence of puromycin. Single-cell clones were selected by limiting dilution. *MTDH* deletion was confirmed by quantitative PCR (qPCR) and by Western blotting. The cells were grown in RPMI-1640/Dulbecco’s modified Eagle’s medium (DMEM) supplemented with 10% fetal bovine serum (FBS and 1% penicillin/streptomycin and maintained at 37 °C in an incubator under an atmosphere containing 5% CO_2_. Cells were routinely screened for the presence of mycoplasma by the University of Iowa DNA Sequencing Core facility.

### Cell viability assays

Cytotoxic effects were determined using the WST-1 method as previously described^31^. Briefly, 10,000 cells per well were seeded into 96-well plates and treated with individual drug for 72 h. Cell viability was evaluated using the cell proliferation reagent WST-1 (Roche, Germany) according to the manufacturer’s protocol. The absorbance of wells was measured with a micro-plate reader (Bio-Rad Laboratories, Hercules, CA, USA).

### Quantitative real-time RT-PCR

Total RNA was isolated using RNeasy Plus Mini kit (Qiagen) and reverse transcribed into complementary DNA (cDNA) using the SuperScript First-Strand Synthesis SuperMix kit (Invitrogen). Quantitative real-time RT-PCR analysis was performed using SYBR Green. The following primers were used:

*MTDH*: 5′-GTAAACGTGATAAGGTGCTGACT-3′

5′-CGGTGGTAACTGTGATGGTATTT-3′

*GPX4*: 5′-ACAAGAACGGCTGCGTGGTGAA-3′

5′-GCCACACACTTGTGGAGCTAGA-3′

*SLC3A2*: 5′-CCAAGGTGAAGGATGCTCTG-3′

5′-TGTGTGACTAGGGATTTTGTATGC-3′

*SLC7A11*: 5′-ATGCAGTGGCAGTGACCTTT-3′

5′-GGCAACAAAGATCGGAACTG-3′

*18S*: 5′-AACTTTCGATGGTAGTCGCCG-3′

5′-CCTTGGATGTGGTAGCCGTTT-3′.

### Crystal violet staining

A total of 1 × 10^5^ cells were grown in 24-well plates and exposed to drugs for 48 h. After the medium was removed, cells were washed in cold phosphate-buffered saline (PBS), fixed with 100% methanol, and stained with 0.5% crystal violet solution.

### Western blotting

Cells were collected and lysed with RIPA buffer (50 mM Tris-HCl, pH 7.4, 150 mM NaCl, 2 mM EDTA, 1% NP-40, 0.1% sodium dodecyl sulfate (SDS), protease inhibitors). Equal amounts of proteins (40 μg) were separated on 10% SDS-polyacrylamide gel electrophoresis gels and then transferred to a nitrocellulose blotting membrane (PALL Corporation, Mexico). After blocking with 5% non-fat milk, the membrane was incubated overnight at 4 °C with the respective primary antibody. The following antibodies were used: anti-c-MYC (1:1000, 13987S), anti-SLC3A2 (1:1000, 13180S), anti-E-cadherin (1:1000, 3195S), and anti-SLC7A11 (1:500, 12691S) from Cell Signaling Technology (Danvers, MA); anti-β-actin (1:10,000, sc-47778), anti-MTDH (1:250, 517220), and anti-GPx4 (1:500, sc-166570) from Santa Cruz Biotechnology; anti-vimentin (1:1000,v6389) from Sigma-Aldrich; anti-ZEB1 (1:1000, NBP1-88845) from Novus Biologicals USA. Membranes were further incubated with secondary antibody (1:10,000, 7074S or 7076S, Cell Signaling Technology) at room temperature for 2 h. Signal bands were detected using the Bio-Rad ChemiDoc system and densitometry were analyzed with Bio-Rad Image Lab Software (Bio-Rad Laboratories).

### RNA-binding protein immunoprecipitation

Magna RIP^TM^ (RNA-binding protein immunoprecipitation) kit (Millipore, Bedford, MA) and real-time PCR were used to confirm the association between *MTDH* with *SLC3A2* and *SLC7A11* as per the manufacturer’s protocol as previously described^13^. Magna RIP^TM^ kit and microarray were used to pull down MTDH-associated RNAs by a rabbit antibody against MTDH (a.a. 315–461, HPA010932, Sigma) and to identify mRNAs that associate with MTDH. Microarray data have been deposited at GEO (www.ncbi.nlm.nih.gov/geo) under accession number GSE30588. SLC3A2 was detected by quantitative real-time RT-PCR.

### Flow cytometry

Cells were seeded in 6-well plates in DMEM or RPMI-1640, supplemented with 10% FBS, and treated with defined drugs for 16 h. Cells were then incubated with 10 μM BODIPY 581/591 C11 (Life Technologies) in the dark for 30 min at 37 °C. After incubation, cells were trypsinized, washed twice with PBS, and re-suspended in 300 μL phenol red-free DMEM with 0.2% bovine serum albumin. Flow cytometry was performed using the BD FACS Canto II equipped with a 488 nm laser for excitation; data were collected using the 530/30 nm band-pass filter.

### GSH assay

Parental cells and *MTDH* KO cells were plated in 100-mm dishes and allowed to grow for 24 h. Cells were then washed with cold PBS and scraped into 300 µL 5% 5-sulfosalicylic acid (Sigma) in water and stored at −20 °C for a maximum of 72 h. Total GSH content was determined as described previously^32,33^. GSH disulfide (GSSG) was determined by adding 35 μL of a 1:1 mixture of 2-vinylpyridine and ethanol to 175 μL of sample and incubating for 2 h before assaying. The rates of the reaction were compared with similarly prepared GSH and GSSG standard curves. GSH determinations were normalized to the protein content of the insoluble pellet from the 5-sulfosalicylic acid dissolved in 2.5% SDS in 0.1 N bicarbonate using the BCA Protein Assay kit (Thermo Scientific).

### GPx4 activity assay

Cells were grown in DMEM media with 10% FBS, and then in 300 ng/mL of sodium selenite for 24 h. Cells were treated with the compound at the indicated concentration in media for 16 h, then harvested by scratch, and finally pelleted by centrifugation. GPx4 activity was determined as described previously^34^. The activity was measured by a coupled enzymatic assay using GSH, GSH reductase, phosphatidylcholine hydroperoxides (PCOOH), and NADPH. The substrate (PCOOH) was prepared by enzymatic hydroperoxidation of phosphatidylcholine using soybean lipoxidase type IV. The nonspecific NADPH oxidation rate was recorded for 4 min at 340 nm; then, the enzyme reaction was started by the addition of PCOOH (10–30 μM). The rate of specific NADPH oxidation was recorded every 20 s for 4 min at 340 nm using a Beckman DU-70 spectrophotometer. The activity was calculated by subtracting the nonspecific NADPH oxidation rate from the observed NADPH oxidation rate after the substrate addition. The specific activity is expressed as milliunits per milligram total cell protein; one unit of enzyme activity catalyzes the oxidation of 1 μM of NADPH per minute.

### Metabolomics assay

The metabolomics assay was performed with the mass spectrometer through our Metabolomics Core Facility (Iowa City, IA). The Metabolomics Core of the Fraternal Order of Eagles Diabetes Research Center provides metabolite profiling using high-resolution mass spectrometry (MS) following gas chromatography (GC). The semi-targeted high-resolution GC-MS protocol can identify and measure more than 100 metabolites, including glycolysis and TCA cycle, neurotransmitters, amino acids, carbohydrates, and fatty acids. One well of a nearly confluent 6-well plate provides enough sample to obtain full metabolite coverage. Six replicates of parental and *MTDH* KO MDA-MB-231 cells were analyzed. Whole cells were washed twice on plates with ice-coldPBS, and then twice with ice-cold water. Cells were frozen by floating the plate on liquid nitrogen.

### Tumor xenograft model and assessment of GPx4 inhibitors RSL3 and ML162, and their combination with sorafenib in vivo

All animal studies were performed under animal protocols #7051085 approved by the University of Iowa Institutional Animal Care and Use Committee (Iowa City, IA).

To generate tumor xenograft models, 5 × 10^6^ *MTDH* WT and KO MDA-MB-231 cells were injected into the second and fifth mammary fat pads (both sides, total four sites) of the NOD.Cg-*Prkdc*^*scid*^ *Il2rg*^*tm1Wjl*^*/*SzJ (NSG, Jackson Laboratories, Bar Harbor, ME) immunodeficient female mice. To study the metastasis from this orthotopic mouse model, tumor volumes were allowed to grow to ~1000 mm^3^, after which livers were resected to examine incidence as well as tumor burden of liver metastasis. To study the expression level of *SLC3A2* and *GPX4* under alternative *MTDH* status (WT vs. KO), xenografted tumors were resected upon sacrifice, followed by tissue fixation, sectioning, and immunohistochemistry (IHC).

To test the antitumor effect of GPx4 inhibitor RSL3, and its combination with system x_c_^−^ inhibitor sorafenib, 5 × 10^6^ MDA-MB-231 cells were implanted on the right flanks of the mice. Once the tumors became palpable, mice were treated with either solvent (control, *n* = 4) or intratumoral RSL3 (100 mg/kg, 2 times per week, *n* = 5), or sorafenib via oral gavage (20 mg/kg, once every day, *n* = 5), or the combination of RSL3 and sorafenib (*n* = 5) for 20 days. Tumor volume and body weight were measured periodically twice per week. Tumor volume was calculated using the formula length x width^2^/2.

To test the combination of GPx4 and system x_c_^−^ inhibitors under alternative *MTDH* status (WT vs. KO), NSG mice were injected with 5 × 10^6^ MDA-MB-231 cells expressing either WT or KO *MTDH* into one of the fifth mammary fat pads (left). Mice were then started on treatment with 50 mg/kg ML162 at the same site one day later. The ML162 was administered locally twice per week for 2 weeks. Sorafenib was given by gavage daily (20 mg/kg) for 2 weeks. Mice were sacrificed after 3 weeks and tumors were collected.

### IHC staining and analysis

Immunostaining was performed using 5-μm-thick sections of 4% paraformaldehyde-fixed paraffin-embedded tissue samples. Immunohistochemical staining for GPx4, MTDH, and SLC3A2 expression were performed on paraffin sections using a rabbit anti-GPx4 antibody (1:250; no. 3649-1; Epitomics). For secondary antibodies, a biotinylated rabbit anti-rat immunoglobulin G (IgG) (1:200; BA-4000; Vector Laboratories, Linaris, Wertheim-Bettingen) or a biotinylated goat anti-rabbit IgG (1:200; BA-1000; Vector Laboratories) was used. Staining was performed using the Vector ABC kit and Vector DAB kit (Vector Laboratories).

### Statistical analysis

Two-way analysis of variance was used for comparisons between control and treatment over a range of doses or times. Median weights of the resected tumors were compared using two-sided paired *t* tests and presented using box-and-whisker plots. All *P* values were two-sided and <0.05 were considered statistically significant.

## Results

### Mesenchymal-high cancers exhibit enhanced sensitivity to ferroptosis inducers

**Fig. 1:**
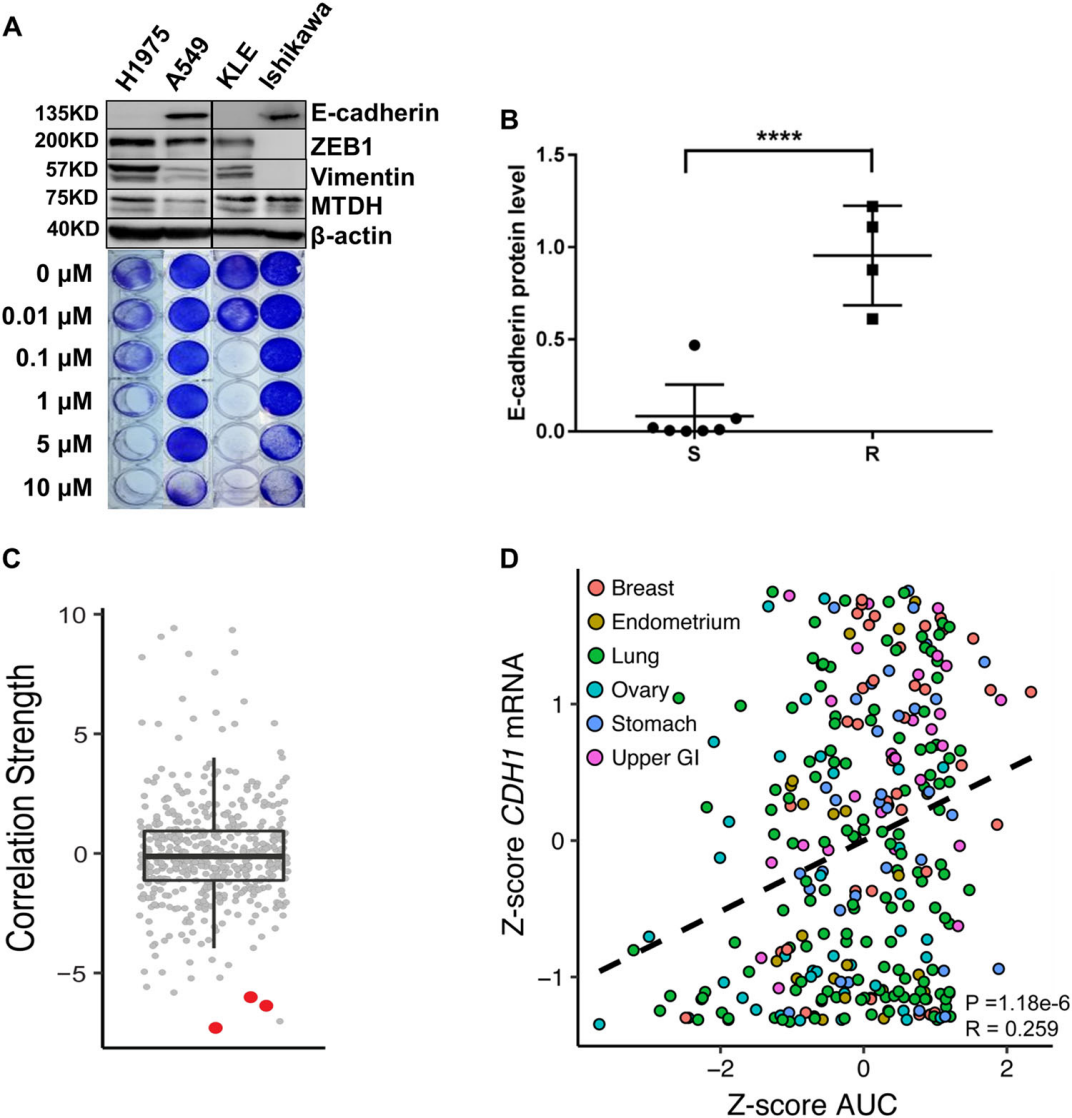
Mesenchymal-high cancers exhibit enhanced sensitivity to ferroptosis inducers. **a** Representative cell lines showing enhanced sensitivity to GPx4 inhibitor ML162 correlates to low levels of E-cadherin, an epithelial marker. H1975 and A549: lung cancer; KLE and Ishikawa: endometrial cancer. Antibodies against E-cadherin, ZEB1, vimentin, and MTDH were used to detect protein extracts from non-small-cell lung cancer H1975 and A549, and endometrial cancer cell lines KLE and Ishikawa. β-Actin was detected as a loading control. **b** Summary of ML162 sensitivity in correlation to E-cadherin expression (R: resistant, *n* = 4; S: sensitive, *n* = 7; ****P < 0.0001 by two-sided paired *t* test). Refer to Supplementary Table 1 for details. **c** *Z*-score-transformed Pearson’s correlations between compound AUCs and the mesenchymal score from single-sample GSEA across 481 different compounds and 516 cell lines derived from CTRP. The GPx4 inhibitors ML162, ML210, and RSL3 are highlighted in red. **d** Correlation of *CDH1* expression vs. AUC for erastin, a system x_c_^−^ inhibitor, across carcinoma cell lines. This is visualizing the type of analysis performed in **C**, but at an individual molecule level and using *CDH1* as a proxy for EMT

To confirm previously reported findings that mesenchymal-high cancer cells exhibit enhanced sensitivity to GPx4 inhibitors^6^, we determined the half-maximal inhibitory concentration (IC_50_) of ML162 in different cell lines, including the NSCLC A549, SCLC DMS53 and DMS273, breast cancer MCF-7 and MDA-MB-231, and endometrial cancer KLE, AN3CA, RL95, Hec50, Hec1A, and Ishikawa cell lines. When we used E-cadherin/β-actin ratio to correlate the IC_50_s, we observed that mesenchymal-high (i.e., low E-cadherin/β-actin ratio) cancer cells exhibited significantly enhanced sensitivity (i.e., low IC_50_) to GPx4 inhibitor ML162 (Fig. 1a, b, Supplementary Table 1). Although MTDH level does not correlate perfectly with E-cadherin in Fig. 1a (e.g., KLE vs. Ishikawa), we do observe an association in general when more cell lines are included (Supplementary Fig. 1). In addition, using the CTRP data, we confirmed that a high mesenchymal score^6^ indeed correlates with increased sensitivity (low area under the curve (AUC) concentration) to GPx4 inhibitors ML162, ML210, and RSL3 (Fig. 1c). A similar correlation was observed for erastin, a system x_c_^−^ inhibitor (Fig. 1d).

### MTDH confers therapy-resistant mesenchymal-high cell state

**Fig. 2:**
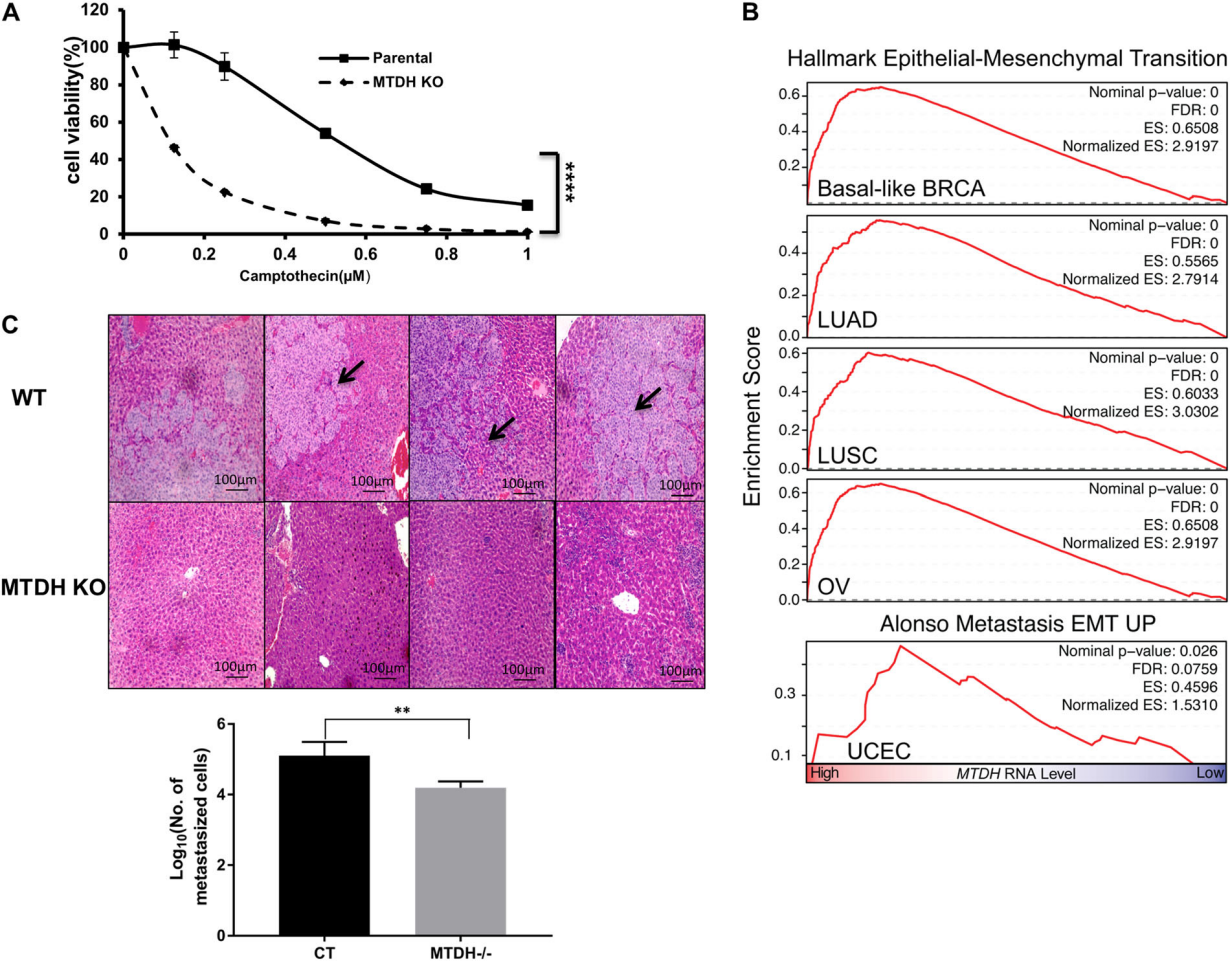
MTDH confers therapy-resistant mesenchymal-high cell state. **a** Sensitivity to the chemotherapeutic agent camptothecin was examined in parental and *MTDH* CRISPR KO Hec50 cells via WST-1 assay. Depletion of MTDH significantly enhanced sensitivity to camptothecin. ****P < 0.0001 by two-way ANOVA. **b** Using TCGA and GSEA *MTDH*-high samples enriched for gene sets involved in EMT across multiple types of cancer. FDR <0.25 considered significant. **c** *MTDH* KO in MDA-MB-231 cells significantly reduced liver metastasis (arrows). **p < 0.01 by two-way ANOVA

As the therapy-resistant mesenchymal-high cell state was found to have enhanced vulnerability to GPx4 inhibitors^5,6^, we determined whether MTDH indeed confers this cell state, since previous studies, including ours, have suggested that MTDH could induce resistance to various treatments including chemotherapy, targeted therapy, and radiation therapy^13,15–18^. Using isogenic Hec50 cells, we observed that *MTDH* depletion (KO) significantly enhanced the sensitivity to the chemotherapeutic agent camptothecin, a topoisomerase inhibitor (Fig. 2a).

To confirm whether MTDH confers a mesenchymal-high cell state, we first performed GSEA using TCGA across multiple cancer types. As shown in Fig. 2b, *MTDH* correlates positively with the gene sets enriched in either EMT or metastasis in various types of cancer, including basal-like breast cancer, lung adenocarcinoma, lung squamous carcinoma, and endometrial cancers. To confirm the causal-effect relationship, we established an orthotopic breast cancer model using isogenic MDA-MB-231 cells with alternative *MTDH* status (WT vs. KO). We found that MTDH depletion decreased the tumor burden of liver metastasis by both IHC (Fig. 2c, upper panel) and a qRT-PCR-based approach, using human α-satellite DNA as we have previously described (Fig. 2c, lower panel)^35^.

### MTDH confers enhanced sensitivity to ferroptosis inducers in vitro

**Fig. 3:**
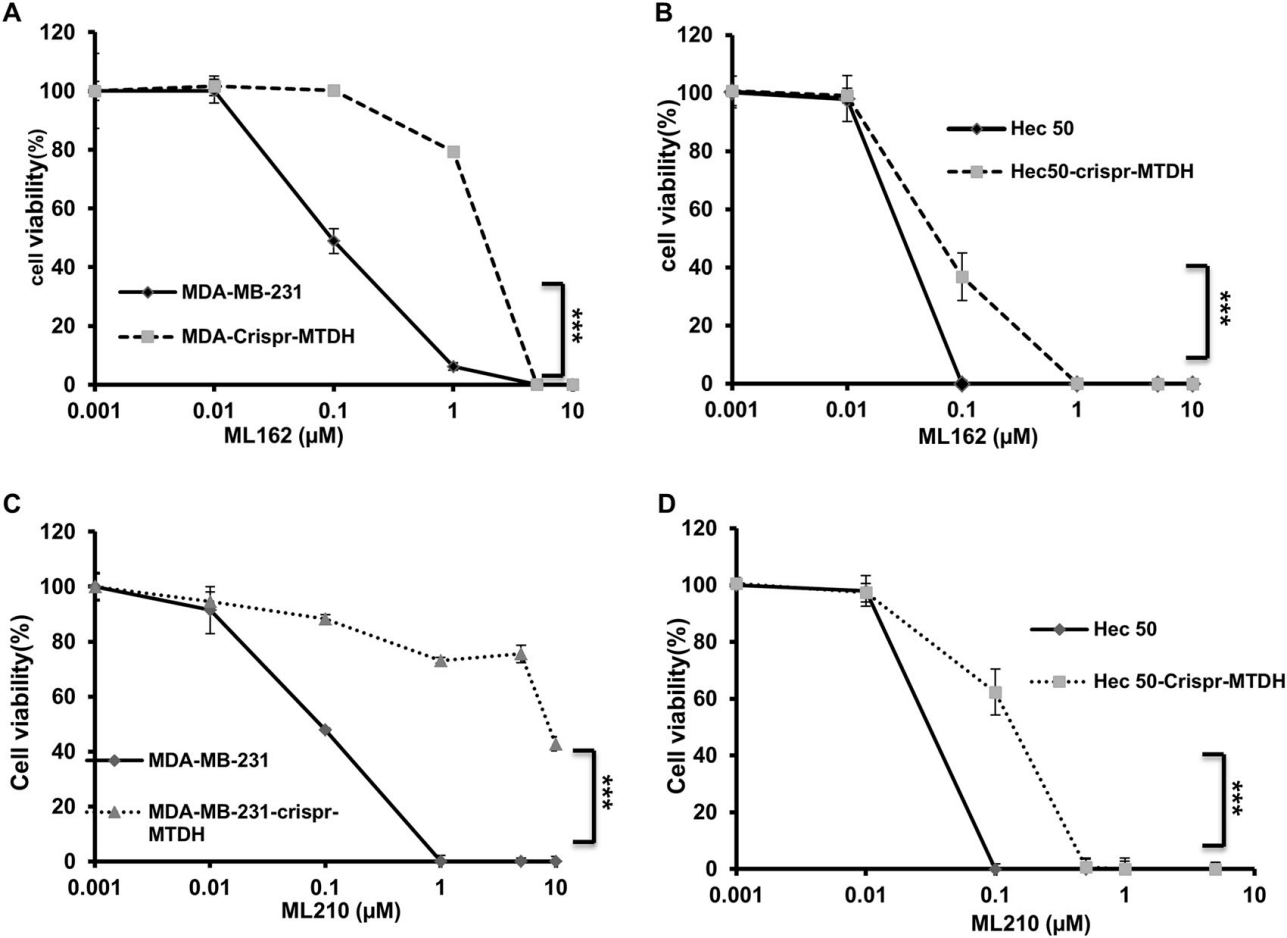
MTDH confers enhanced sensitivity to ferroptosis inducers in vitro. **a**, **b** ML162. **c**, **d** ML210. **a**, **c** MDA-MB-231. **b**, **d** Using Hec50. In all cases, as compared to *MTDH* KO cells, *MTDH*-high WT cells exhibited enhanced sensitivity to GPx4 inhibitors. WT: wild type; KO: CRISPR knockout isogenic cells

Since therapy-resistant mesenchymal-high cancers have increased vulnerability to ferroptosis inducers, especially GPx4 inhibitors^5,6^, and MTDH promotes EMT and treatment resistance, we therefore investigated whether MTDH confers enhanced vulnerability to ferroptosis. Indeed, in both MDA-MB-231 and Hec50 cells, knocking out *MTDH* significantly reduced their sensitivity to GPx4 inhibitors ML162 and ML210 (Fig. 3a–d).

### MTDH downregulates GPx4 and SLC3A2

**Fig. 4:**
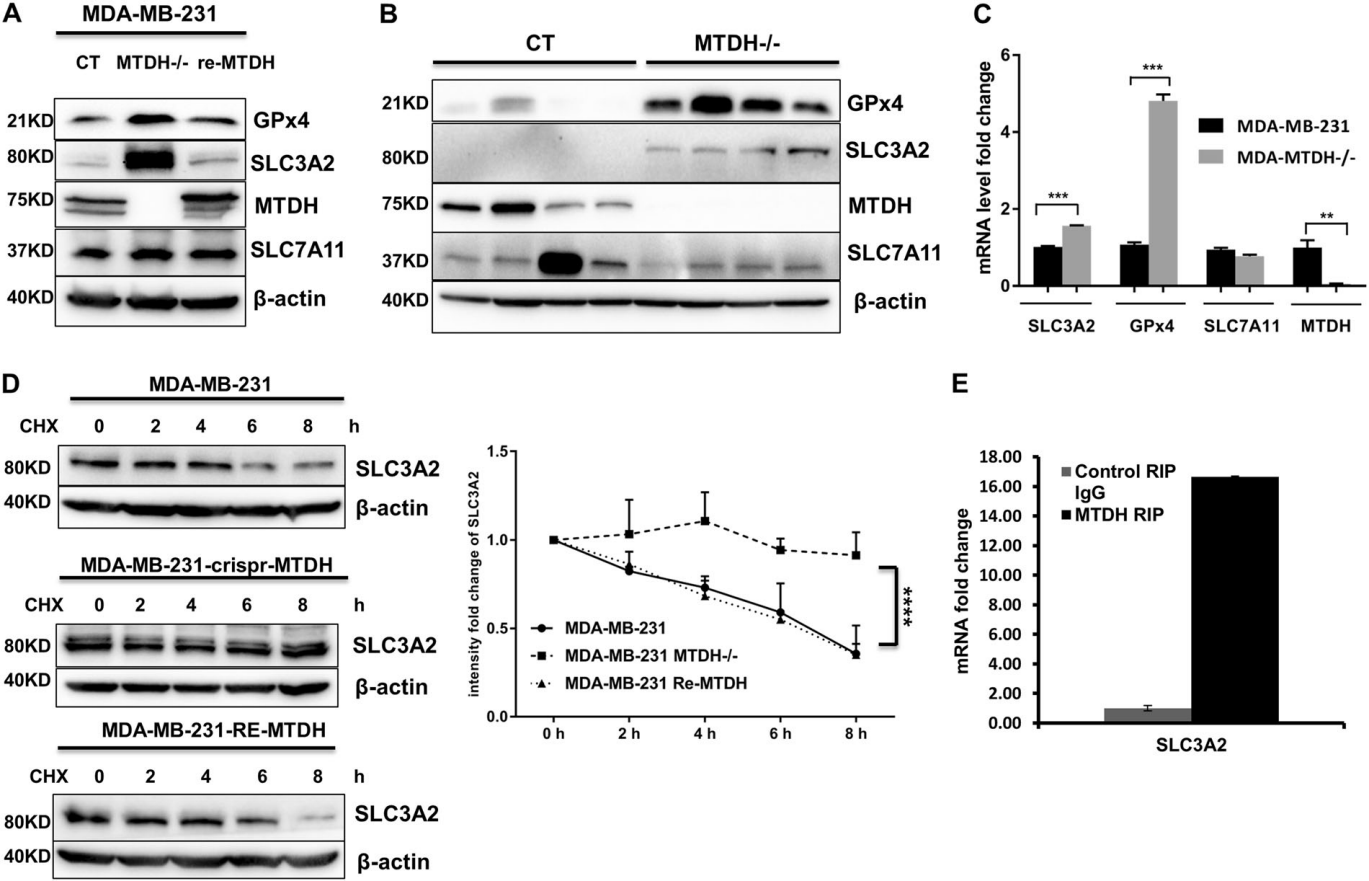
MTDH downregulates GPx4 and SLC3A2. **a** Using MDA-MB-231 cells, knocking out *MTDH* increased protein levels of SLC3A2 and GPx4, which were reversed by re-expression of *MTDH*. **b** Such regulation holds in vivo. *MTDH* WT and KO MDA-MB-231 cells were implanted into NSG mice (four mice each group), and tumors were harvested to check expression levels of SLC3A2 and GPx4. CT: control using WT cells. **c**. Knocking out *MTDH* also increased mRNA levels of *SLC3A2* and *GPX4* in MDA-MB-231 cells. **P < 0.01, ***P < 0.001 by two-way ANOVA. **d** Effect of MTDH on protein stability of SLC3A2. *MTDH* WT, KO and re-expresser of MDA-MB-231 cells were treated with 20 µg/mL cycloheximide (CHX) for 0, 2, 4, 6, and 8 h. Cell lysates were then subjected to Western blot analysis with anti-SLC3A2 and anti-β-actin antibodies. Densitometric quantification was used and normalized to loading control. Data are a representative of three independent experiments. ****P < 0.0001 by two-way ANOVA. **e** RIP assay. Compared to control IgG, MTDH-specific antibody resulted in dramatically increased pull-down yield of the mRNA of *SLC3A2*

To understand how MTDH enhances vulnerability to ferroptosis, we examined expression levels of the key members involved in the lipid peroxidation pathway. We found that *MTDH* KO upregulated the expression of SLC3A2 and GPx4 (Fig. 4a). Importantly, when we re-expressed *MTDH* in *MTDH* KO MDA-MB-231 cells, we observed a downregulation of both SLC3A2 and GPx4 (Fig. 4a), suggesting that MTDH directly regulates SLC3A2 and GPx4, and therefore contributes to the enhanced vulnerability to ferroptosis. Interestingly, no significant change was observed for SLC7A11 under alternative *MTDH* status (Fig. 4a). To confirm whether such regulation occurs in vivo, we injected *MTDH* WT and KO MDA-MB-231 cells into the mammary fat pad of immunodeficient female mice, and again we observed that the tumors which developed from *MTDH* WT cells exhibited reduced levels of SLC3A2 and GPx4, which was confirmed via both western blot (Fig. 4b) and IHC (Supplementary Fig. 2).

Similarly, at the mRNA level, we observed statistically significant upregulation of both *SLC3A2* and *GPX4* when *MTDH* was depleted (Fig. 4c). Interestingly, the change in mRNA levels of *SLC3A2* was less dramatic compared to the change at the protein level, suggesting that additional post-transcriptional mechanisms are involved. We therefore examined the effect of MTDH on the protein stability of SLC3A2 using cycloheximide. As shown in Fig. 4d, knocking out *MTDH* significantly prolonged the half-life of SLC3A2, which was again shortened to about 6 h after re-expressing *MTDH*. In addition, since we recently discovered that MTDH can function as an RNA-binding protein to regulate the translation of other genes^13^, we questioned whether this could be an additional mechanism of regulation. Such speculation is supported by a recent study suggesting that *MTDH* possesses putative binding sites on the mRNA of multiple key members of the antioxidant system, including SCL3A2, SLC7A11, and GPx4 (Supplementary Table 2)^36^. To confirm the mRNA-binding capacity of MTDH, we performed a pilot study using RIP. Compared to the control IgG, we found that the MTDH-specific antibody dramatically increased the pull-down yield of the mRNA of *SLC3A2* (Fig. 4e). This suggests that MTDH is capable of binding to *SLC3A2* mRNA, and the regulation of SLC3A2 by MTDH is through multiple mechanisms at both the transcriptional and post-transcriptional levels.

### MTDH induces metabolomic changes that increase the susceptibility to ferroptosis

**Fig. 5:**
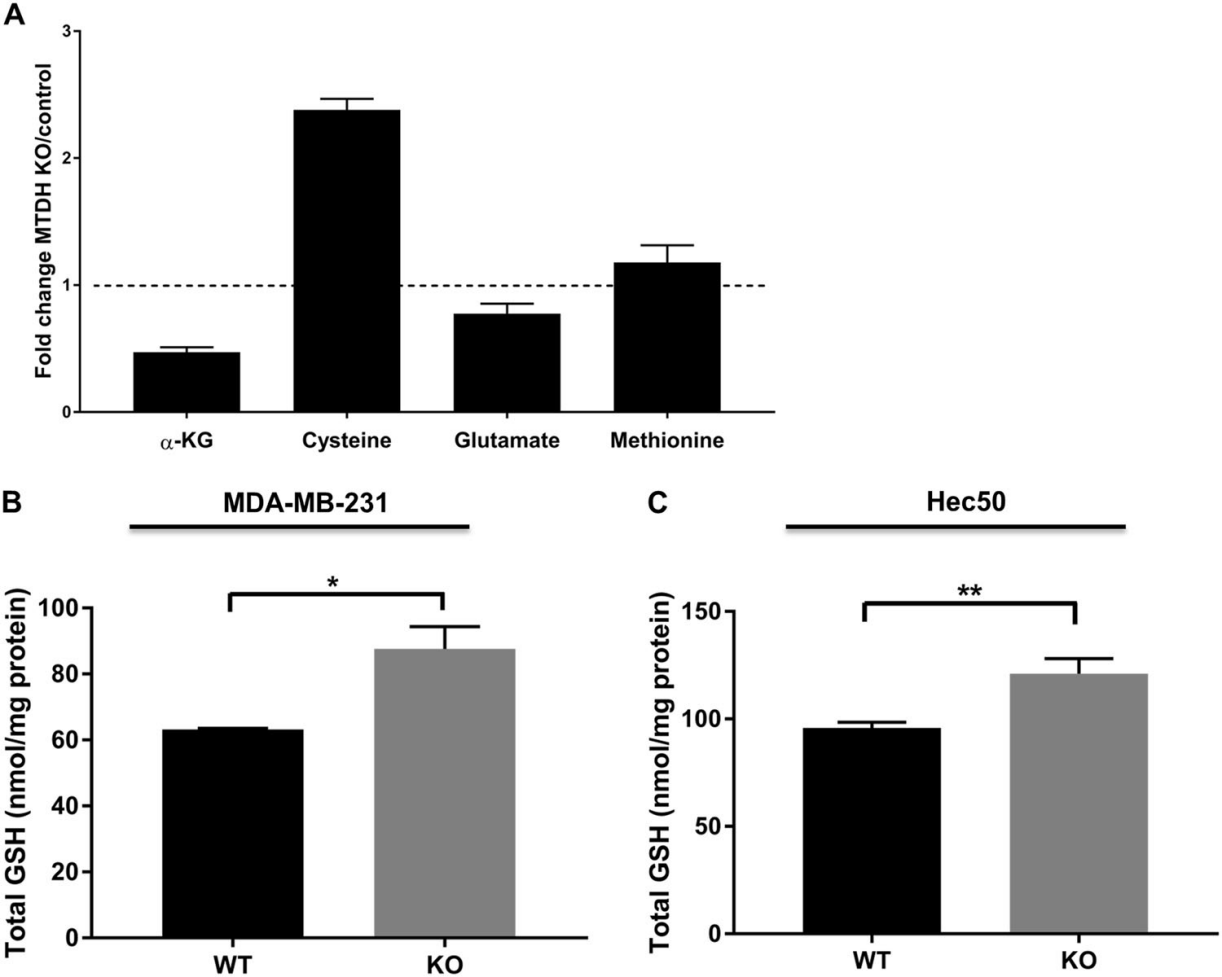
MTDH induces metabolomic changes that increase the susceptibility to ferroptosis. **a**. Using the MDA-MB-231 cell line, knocking out *MTDH* increased intracellular cysteine and methionine, but reduced glutamate and α-KG levels. **b**. *MTDH* KO in MDA-MB-231 cells resulted in elevated levels of glutathione. **c**. Similar results were observed in Hec50 cells. *P < 0.05, **P < 0.01 by two-way ANOVA

As the downregulation of SLC3A2 by MTDH could potentially impair the function of system x_c_^−^, we questioned whether MTDH could reduce intracellular cysteine and increase glutamate levels, which will eventually lead to reduced levels of GSH, or whether *MTDH* KO will have the opposite result. We therefore performed a metabolomics study using isogenic MDA-MB-231 with alternative *MTDH* status (WT vs. KO). We observed that *MTDH* KO dramatically increased cysteine levels (Fig. 5a, Supplementary Table 3). In addition, methionine, which can be used to synthesize cysteine via the *trans*-sulfuration pathway, also increased after knocking out *MTDH* (Fig. 5a). In contrast, the level of glutamate was reduced in *MTDH* KO cells. This is supported by the reduced level of α-ketoglutarate (Fig. 5a), a product of glutaminolysis that is required for ferroptosis^37^. Therefore, *MTDH* KO can protect cells from ferroptosis. This was further confirmed via measurement of GSH levels; knocking out *MTDH* in both MDA-MB-231 and Hec50 cells increased levels of GSH significantly (Fig. 5b, c). These data suggest that MTDH attenuates system x_c_^−^ function (or *MTDH* KO improves), which could theoretically improve cell viability under glucose-deficient/glutamine-replete conditions due to enhanced ability to use intracellular glutamate to maintain respiratory chain activity^38^.

### Inhibition of GPx4 has the strongest effect in inducing ferroptosis, and enhances the tumor-suppressing function of other ferroptosis inducers in vitro

**Fig. 6:**
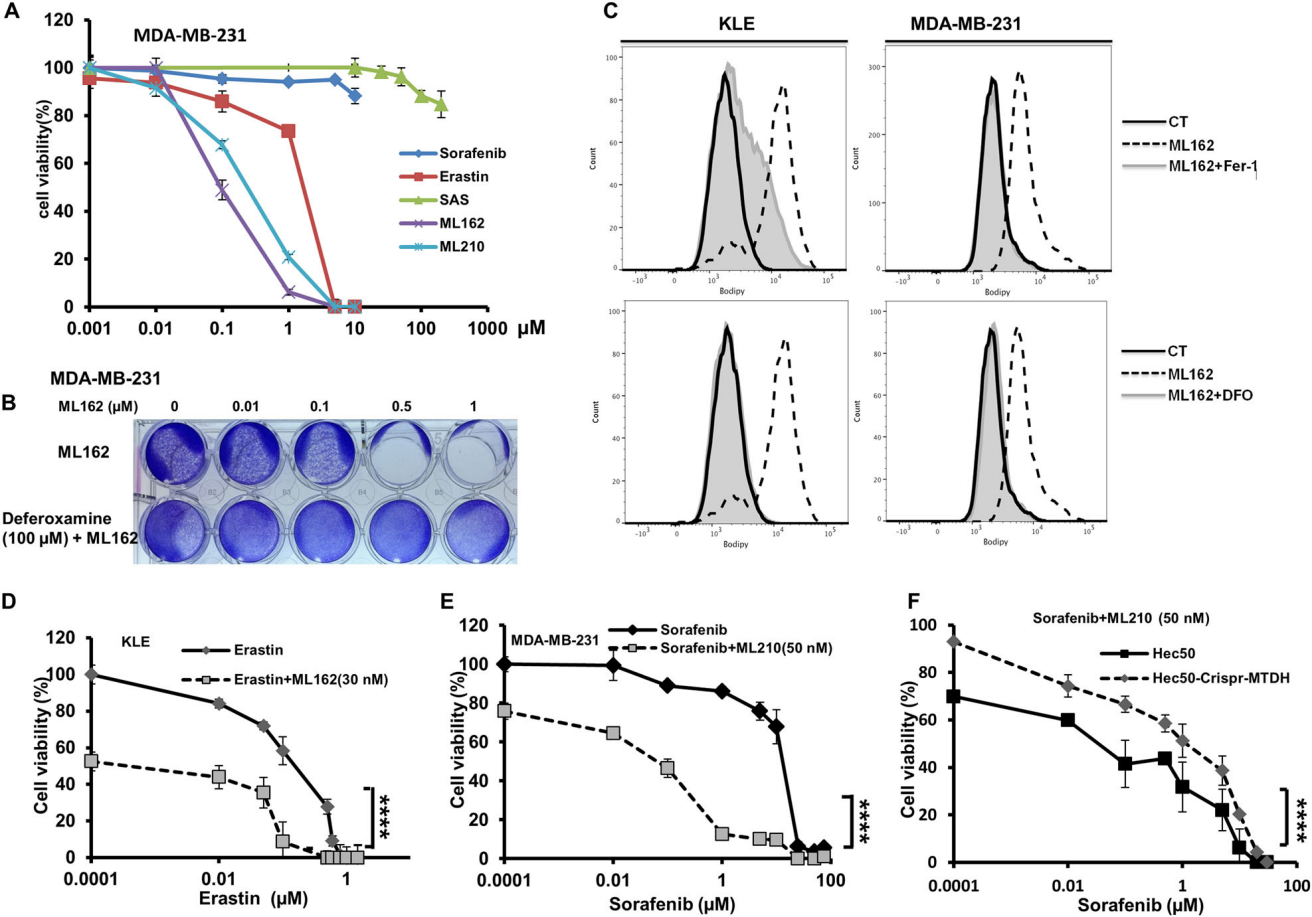
GPx4 inhibition has the strongest effect in inducing ferroptosis, and enhances tumor-suppressing function from other ferroptosis inducers in vitro. **a** Using the MDA-MB-231 cell line as an example to compare various ferroptosis inducers. **b** Deferoxamine (an iron chelator) completely blocked the cytotoxic effect of ML162 in MDA-MB-231 cells, suggesting that the effect was enhanced through the induction of ferroptosis. **c** Treatment of cells with ML162 leads to increased lipid peroxidation as assessed by C11-BODIPY 581/591 fluorescence in KLE and MDA-MB-231 cells. Co-treatment with the lipophilic antioxidant ferrostatin-1 (Fer-1) or deferoxamine almost completely blocked ML162-induced lipid peroxidation. **d** Using KLE cells to compare erastin vs. erastin plus ML162 (30 nM). ****P < 0.0001 by two-way ANOVA. **e** Using MDA-MB-231 cells to compare erastin vs. erastin plus ML210 (100 nM). ***P < 0.001 by two-way ANOVA. **f** Using *MTDH* WT vs. KO Hec50 cells to compare the combination of sorafenib plus ML210 (50 nM). ***P < 0.001 by two-way ANOVA

Using multiple cells lines, we tested various ferroptosis inducers, including: the system x_c_^−^ inhibitors erastin, sulfasalazine, and sorafenib; buthionine sulfoximine, which depletes GSH; and statins that are indirect inhibitors of GPx4; as well as direct GPx4 inhibitors RSL3, ML162, and ML210. Overall, GPx4 inhibitors exhibit the lowest half-maximal effective concentration (EC_50_) (i.e., most potent) (Fig. 6a, Supplementary Fig. 3). To confirm whether the cytotoxic effect of GPx4 inhibitors was occurred through ferroptosis, we tested the iron chelator deferoxamine. We found that deferoxamine fully blocked or prevented cytotoxicity (Fig. 6b, Supplementary Fig. 3A). In addition, using C11-BODIPY (581/591), a fluorescent probe for lipid peroxidation, we found that treatment with ML162 in both the KLE and MDA-MB-231 cells led to increased lipid peroxidation that could be completely blocked by either lipophilic antioxidant Fer-1 or deferoxamine (Fig. 6c, Supplementary Fig. 4). To confirm the effect on GPx4 activity by ML162, we also measured GPx4 activity in multiple endometrial and breast cancer cell lines after treatment with ML162. We found that GPx4 activity could be reduced by more than 50% with just 0.1 µM of ML162 (Supplementary Fig. 5). Further exploration in the therapeutic value of the inhibition of GPx4 should have high priority among all ferroptosis inducers.

Since the activity of GPx4 relies on GSH, we therefore tested whether GPx4 inhibitors and other ferroptosis inducers can enhance each other in killing cancer cells. As an example, we tested erastin in combination with ML162 in KLE cells (Fig. 6d), and erastin and ML210 in MDA-MB-231 cells (Fig. 6e), as well as sorafenib and ML210 in Hec50 cells with alternative *MTDH* status (WT vs. KO, Fig. 6f). We found that a small dose of GPx4 inhibitor drastically increased growth inhibition from other ferroptosis inducers. In addition, we found that compared to the WT cells, both *MTDH* KO Hec50 (Fig. 6f) and MDA-MB-231 cells (Supplementary Fig. 3J) were more resistant to the combination of ML210 and sorafenib, which is consistent with our observation that MTDH confers sensitivity to ferroptosis inducers (Fig. 3).

### MTDH levels correlate with the ferroptotic effect

**Fig. 7:**
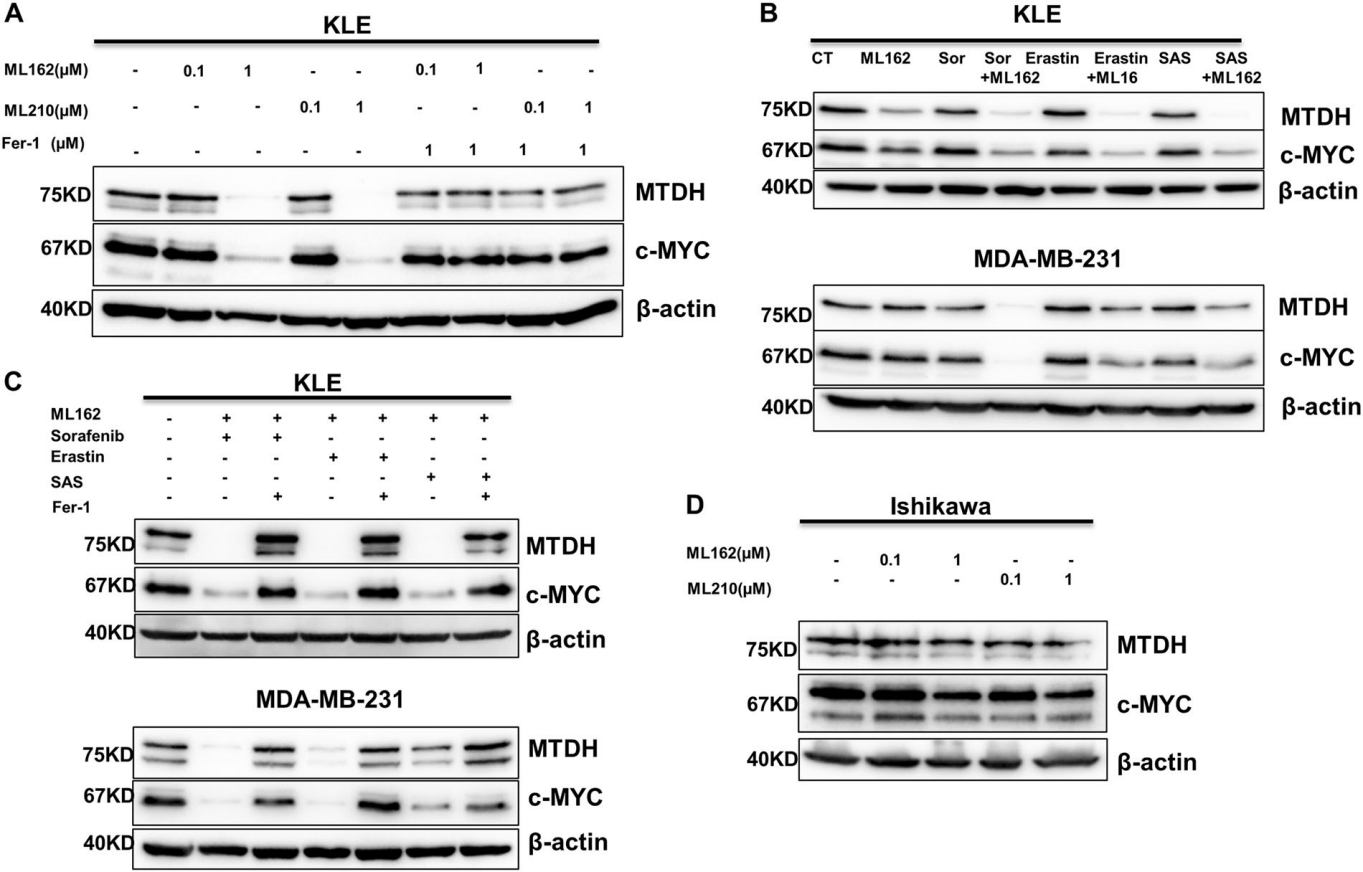
MTDH levels correlate with the ferroptotic effect. **a** Using KLE cells, MTDH levels were reduced with increasing doses of ML162 and ML210, which could be reserved by ferroptosis inhibitor ferrostatin-1 (1 µM). **b** Similar changes were observed when ML162 was used in combination with other ferroptosis inducers (sorafenib: 5 µM; erastin: 2.5 µM; SAS: 400 µM) in MDA-MB-231 and KLE cells. **c** MTDH levels were reserved by ferrostatin-1 (1 µM) with a combination treatment. **d** However, such correlation between MTDH levels and dose of ferroptosis inducers were not observed in ferroptosis-resistant Ishikawa cells

Since MTDH confers enhanced vulnerability to ferroptosis inducers, we questioned whether levels of MTDH correlate to the ferroptotic effect, and therefore might serve as a biomarker for ferroptotic cell death. In fact, using MTDH-high KLE and MDA-MB-231 cells, we observed that MTDH levels were reduced with increasing doses of GPx4 inhibitors ML162 and ML210, which could be completely reversed by the ferroptosis inhibitor Fer-1 (Fig. 7a). This was again observed when different ferroptosis inducers were used in combination in both KLE and MDA-MB-231 cells, which also could be reversed by Fer-1 (Fig. 7b, c). Interestingly, in ferroptosis-resistant Ishikawa cells, the reduction of MTDH expression was not observed (Fig. 7d). These observations therefore suggest the potential role of MTDH as a therapeutic biomarker for ferroptosis that is worth further exploration.

### Inhibitors of GPx4 enhance the antitumor effect of inhibitors of system x_c_^−^ in MTDH-high but not MTDH-null tumors in vivo

**Fig. 8:**
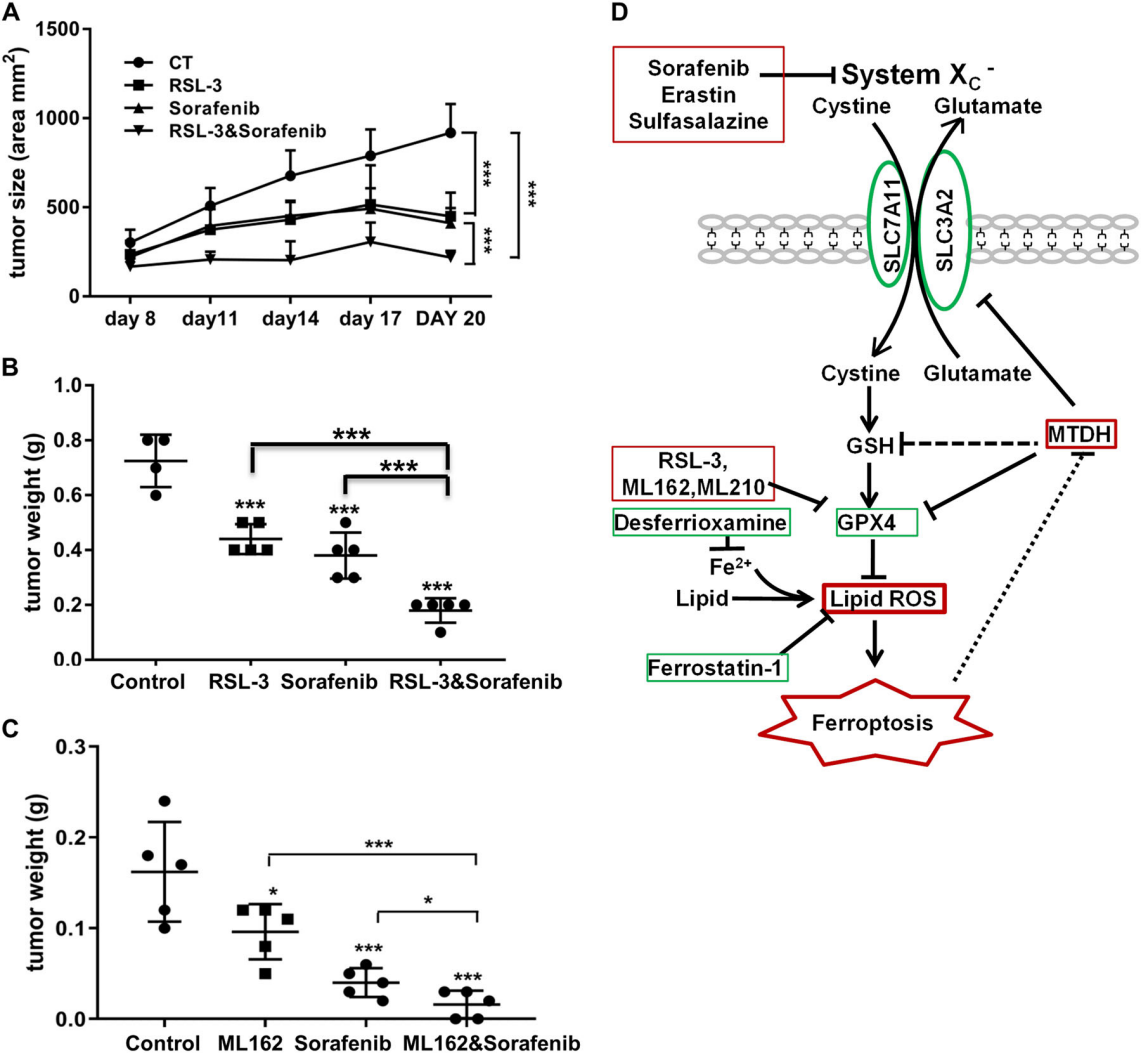
GPx4 inhibitors enhance antitumor effect of system x_c_^−^ inhibitor in MTDH-high tumors in vivo. **a**, **b** Xenograft study using MDA-MB-231 cells expressing WT *MTDH* (high). This is a therapeutic study to test the effect of RSL3 on preexisting tumors. Mice were treated with either vehicle (ctrl, *n* = 4) or intratumoral RSL3 (100 mg/kg, 2 times per week, *n* = 5), or sorafenib via gavage (20 mg/kg, daily, *n* = 5), or the combination of RSL3 and sorafenib (*n* = 5) for 20 days. ***P < 0.001 by two-way ANOVA. **a** Tumor growth curve; **b** tumor weight after sacrifice. **c** A tumor prevention study using ML162 alone or in combination with sorafenib. Treatment was started 1 day after implantation of MDA-MB-231 cells expressing WT *MTDH* (high). ML162 was given by local injection at 50 mg/kg two times per week, and sorafenib was given orally daily. Treatment lasted for 2 weeks, followed by another week of observation before mice were sacrificed and tumors harvested. The combination of ML162 and sorafenib resulted in significantly smaller tumors compared to sorafenib alone (*P* = 0.047). *P < 0.05, **P < 0.01 by two-way ANOVA. **d** A scheme describing the role of MTDH in ferroptosis. Red or green color represents compounds and molecules/proteins that promote or inhibit ferroptosis, respectively. MTDH promotes ferroptosis by downregulating SLC3A2, GPX4, and GSH. All compounds (either in red or green) were tested in this study

As we observed that the GPx4 inhibitors ML162 and ML210 enhanced the tumor killing effect of the system x_c_^−^ inhibitor sorafenib in vitro, we explored whether a similar effect could be observed in vivo. Due to biostability issues, currently available GPx4 inhibitors are not suitable for systemic use^39^; therefore, we performed intratumoral administration as previously described^3^. Using an MDA-MB-231 orthotopic breast cancer model, we found that the combination of the GPx4 inhibitor RSL3 and system x_c_^−^ inhibitor sorafenib resulted in the strongest inhibition of tumor growth and lowest tumor weight (Fig. 8a, b). To test whether a combination effect can be observed using other GPx4 inhibitors in an MTDH-dependent manner, and to prevent tumor growth, we replaced RSL3 with ML162. To the best of our knowledge, this has never been tested in vivo. Treatment was started 1 day after tumor implantation. Again, we observed that ML162 enhanced the antitumor effect of sorafenib in MTDH-high tumors (Fig. 8c). However, this combination effect was not observed in MTDH-null tumors derived from *MTDH* KO cells (Supplementary Fig. 6). These observations therefore suggest the overexpression of MTDH as a therapeutic biomarker for targeting GPX4 and system x_c_^−^.

## Discussion

Since therapy-resistant cancers have been found to have increased vulnerability to ferroptosis^6,7^, finding novel approaches to selectively induce ferroptosis in cancer cells and exploring the underlying mechanisms are gaining more attention.

Although the therapy-resistant mesenchymal-high cell state confers enhanced sensitivity to GPx4 inhibitors^5,6^, the exact underlying mechanism remains to be deciphered. Here we showed that MTDH is one of the plausible key players. Using various cell lines and publicly available CTRP data, we confirmed that mesenchymal-high cancers exhibit enhanced sensitivity to ferroptosis inducers, especially the GPx4 inhibitors. We then confirmed that MTDH confers the therapy-resistant mesenchymal-high cell state. Such findings are consistent with previous observations showing that MTDH can activate both NF-κB^19^ and TWIST1^20^, and therefore promote EMT. This is also supported by our previous studies using IHC on human endometrial cancer samples, which showed that MTDH expression increases with increasing stages of endometrial cancer^17^.

The role of MTDH in treatment resistance, EMT, and metastasis has been documented in previous studies, including ours^14,17,18,21,40^. Here we provide strong supporting evidence from TCGA and GSEA, along with in vitro and in vivo data. Although these observations aligns with other survival advantages provided by MTDH (e.g., enhanced EMT and drug resistance), such advantages are not gained without a price: due to the attenuation of system x_c_^−^ function, MTDH-high cancers exhibit enhanced vulnerability to ferroptosis—a potential “Achilles” heel.” These observations also suggest a role for SLC3A2 in lipid peroxidation and ferroptosis, which is less known compared to its heterodimerization partner SLC7A11. We therefore established the connection between MTDH and lipid peroxidation (Fig. 8d). Our hypothesis that MTDH links to ferroptosis is well supported: using isogenic cell lines with alternative *MTDH* status (WT vs. KO), we observed *MTDH*-high (WT) cells exhibited enhanced vulnerability to GPx4 inhibitors ML162 and ML210. Mechanistically, we found that MTDH downregulated both GPx4 and SLC3A2. Interestingly, its regulation of SLC3A2 was found at both the transcriptional and post-transcriptional levels: aside from reducing the mRNA level of *SLC3A2*, MTDH also negatively affected its protein stability. In addition, the mRNA species of key members of the lipid hydroperoxide removal system have putative MTDH binding sites, including *SLC3A2*, *SLC7A11*, and *GPX4*^36^. We have previously demonstrated that MTDH may act as an RNA-binding protein;^13^ thus, we hypothesized this as an additional mechanism for how MTDH regulates lipid peroxidation. Using RIP assay, we observed that MTDH antibody was able to pull down significantly more mRNA of *SLC3A2* compared to control IgG. In addition, through metabolomics study, we found that MTDH reduced the intracellular concentration of cysteine, but increased glutamate, suggesting a negative regulation of system x_c_^−^, the cystine/glutamate antiporter. This was consistent with our observation of reduced levels of GSH, setting the stage for enhanced ferroptosis^7^, which also suggests a potential role of SLC3A2 in regulating lipid peroxidation.

As GPx4 activity relies on GSH levels, we also tested the combination of GPx4 inhibitors with various other ferroptosis inducers, especially system x_c_^−^ inhibitors. Our in vitro assay suggested a clear combinational effect only in *MTDH* WT, but not *MTDH* KO isogenic cells, again confirming the role of MTDH in ferroptosis.

Although GPx4 inhibitors are the most potent inducers of ferroptosis, all currently reported direct GPx4 inhibitors (e.g., ML162, ML210, and RSL3) have biostability issues that make them unsuitable for systemic use in vivo^3,39^. We therefore tested intratumoral administration of both RSL3 and ML162 as an approach to bypass this application hurdle. Consistent with previous studies^3^, intratumoral RSL3 was effective, and enhanced the antitumor effect of the system x_c_^−^ inhibitor sorafenib, confirming the combinational effect observed in vitro. In addition, intratumoral injection of ML162 also prevented tumor growth alone or combined with sorafenib, suggesting that intratumoral administration could be a feasible delivery approach for direct GPx4 inhibitors. This is clinically important as intratumoral injection is a viable treatment approach for certain cancers at certain locations (e.g., tumors in the trachea). In the era of cancer immunotherapy, intratumoral injections of chemotherapy or biological agents are often explored in combination with systemic anti-PD-1/PD-L1 agents^41,42^. Since antigens released upon cancer cell death may potentiate the immune checkpoint blockade (the rationale behind the combination with chemotherapy in the clinic setting^43,44^), and ferroptosis is an effective form of cell death in apoptosis-resistant cancers^5,6^, intratumoral injection of GPx4 inhibitors might offer a novel strategy to enhance cancer immunotherapy. In fact, this hypothesis is currently being tested in our laboratory. In addition, our in vivo study showed that ferroptosis inducers and their combination were effective only in *MTDH* WT, but not *MTDH* KO tumors, again confirming the value of MTDH in modulating lipid peroxidation.

In summary, the present study demonstrates that MTDH enhances the vulnerability of cancer cells to ferroptosis, and suggests that MTDH may be a therapeutic biomarker for future ferroptosis inducing agents to treat cancer.

## Supplementary information

Supplemental figure 1

Supplemental figure 2

Supplemental figure 3

Supplemental figure 3

Supplemental figure 4

Supplemental figure 5

Supplemental figure 6

Supplemental table 1

Supplemental table 2

Supplemental table 3
