# Supplementary figures and images for: Metadherin enhances vulnerability of cancer cells to ferroptosis
Source: Cell Death Dis. 2019 Sep 17;10(10):682. doi: 10.1038/s41419-019-1897-2 (PMC6746770; doi:10.1038/s41419-019-1897-2)

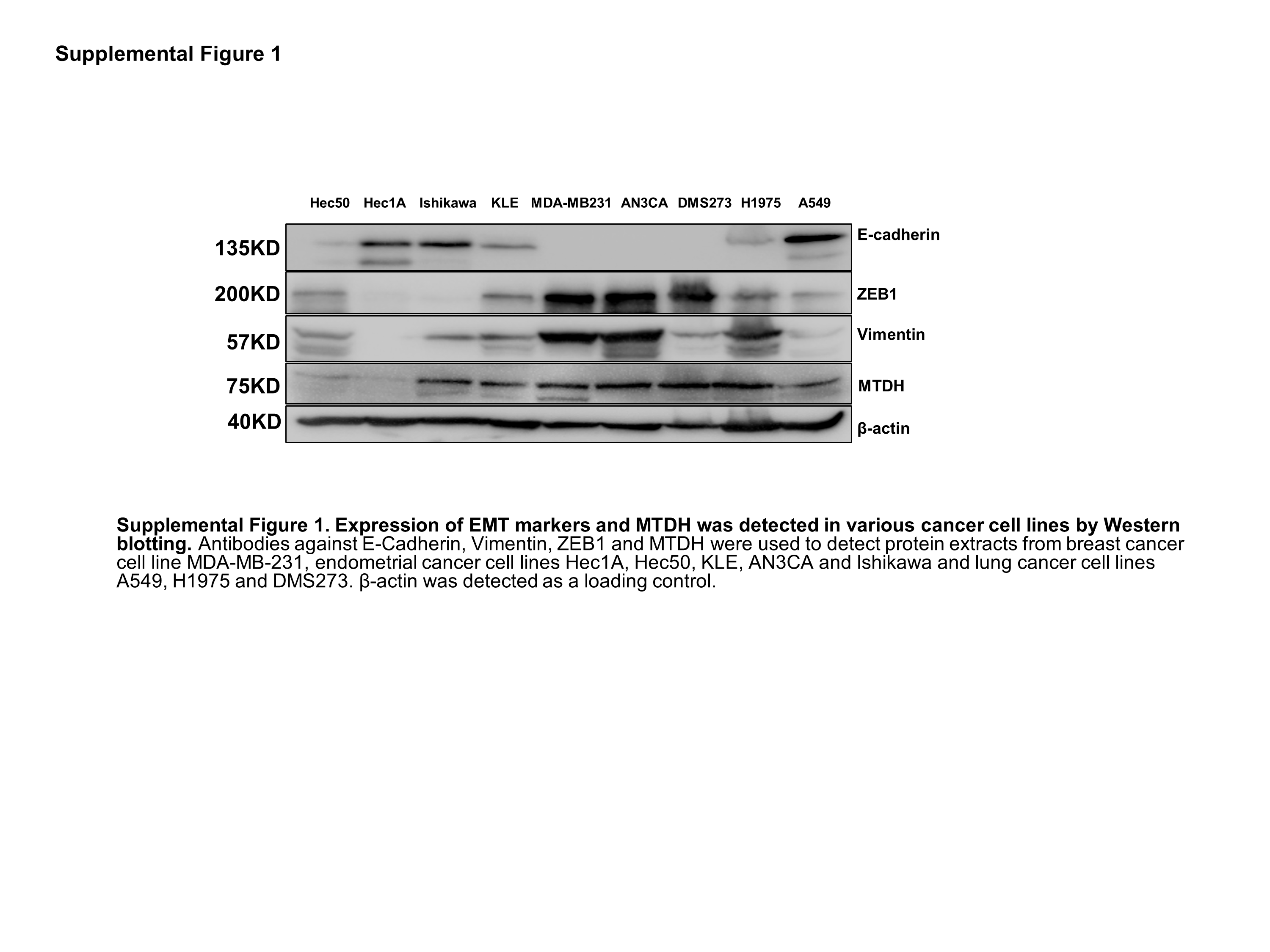

Supplement: Supplementary file 1 — Supplemental figure 1 [file 41419_2019_1897_MOESM1_ESM.tif]

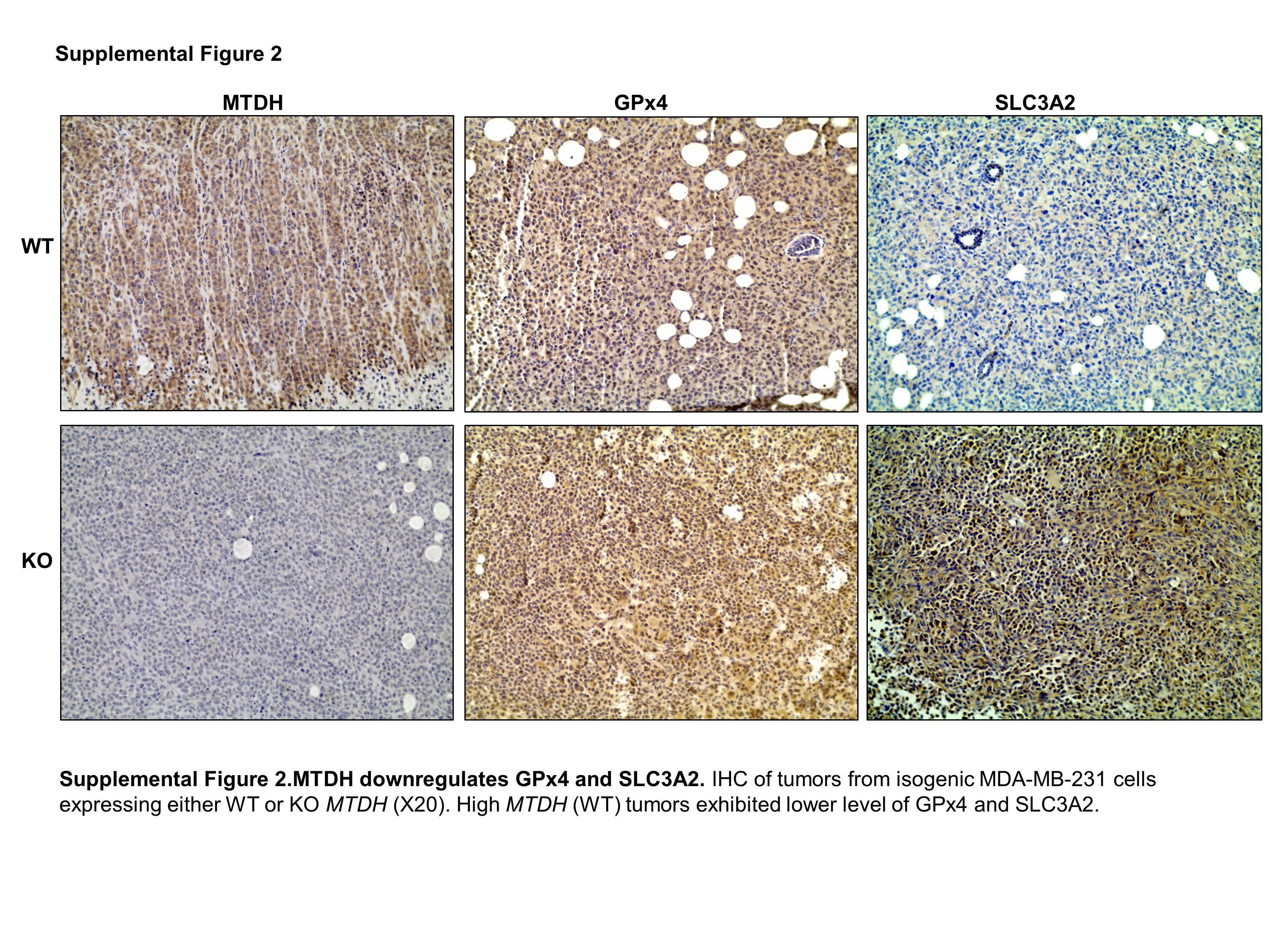

Supplement: Supplementary file 2 — Supplemental figure 2 [file 41419_2019_1897_MOESM2_ESM.tif]

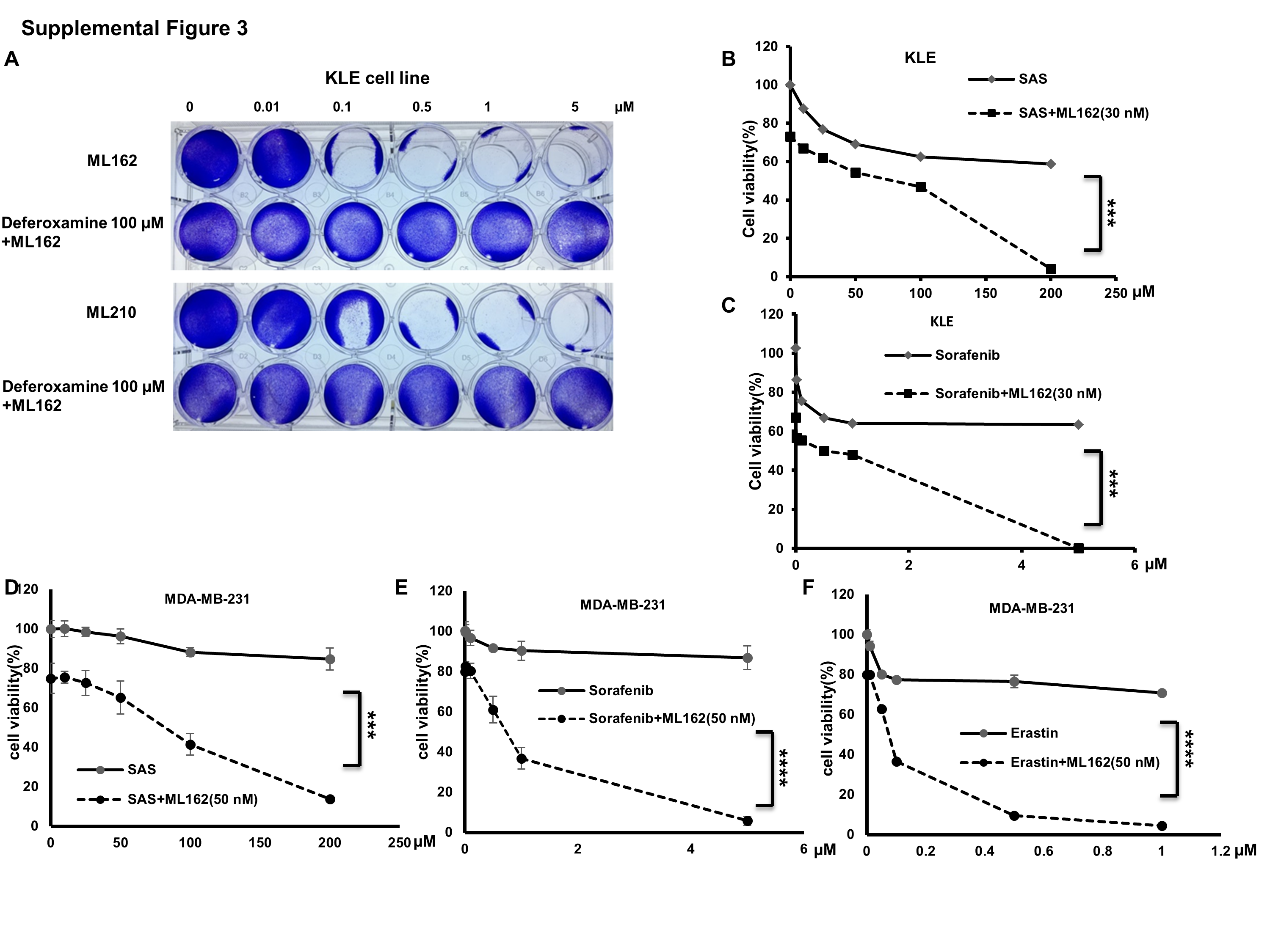

Supplement: Supplementary file 3 — Supplemental figure 3 [file 41419_2019_1897_MOESM3_ESM.tif]

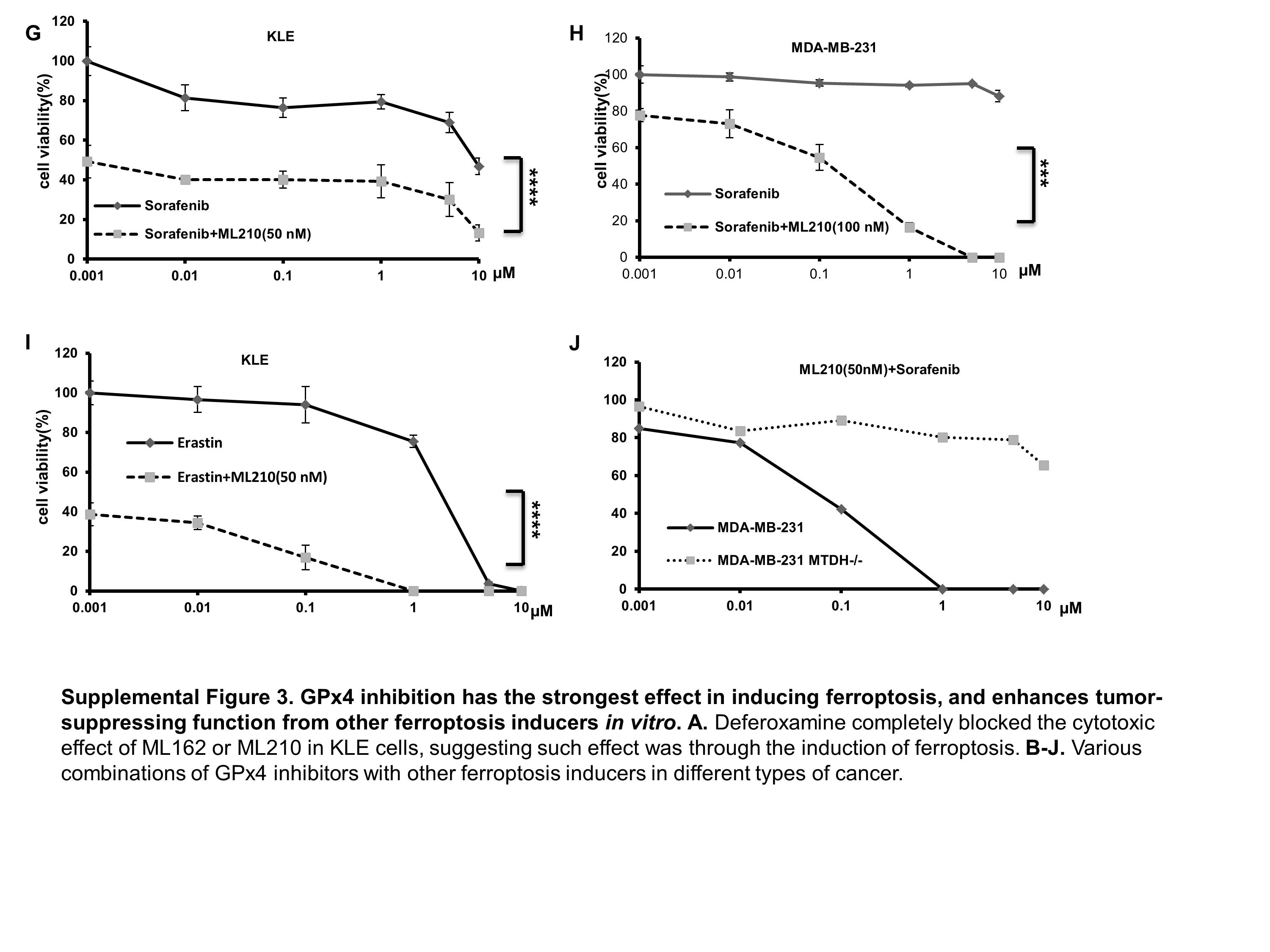

Supplement: Supplementary file 4 — Supplemental figure 3 [file 41419_2019_1897_MOESM4_ESM.tif]

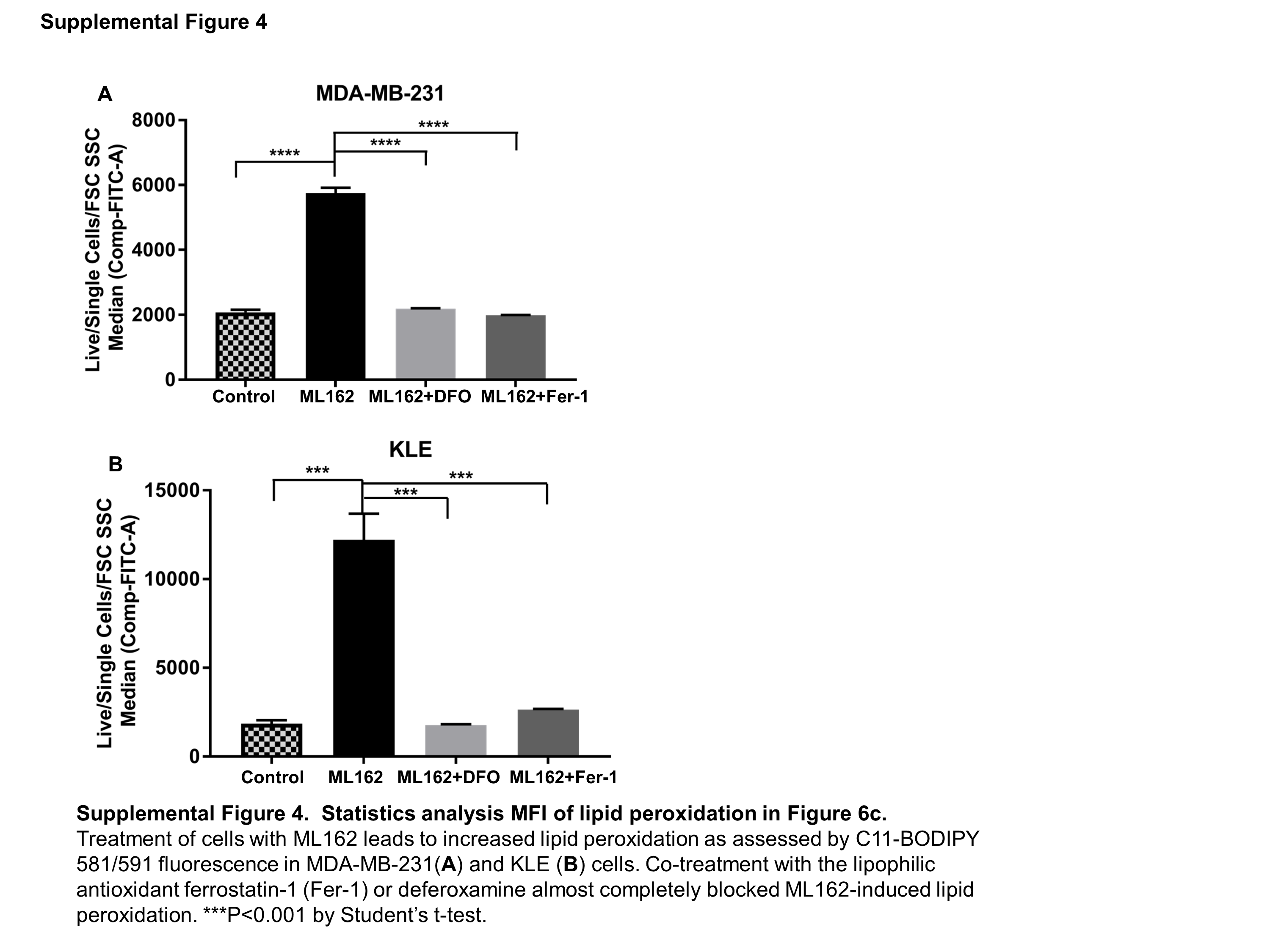

Supplement: Supplementary file 5 — Supplemental figure 4 [file 41419_2019_1897_MOESM5_ESM.tif]

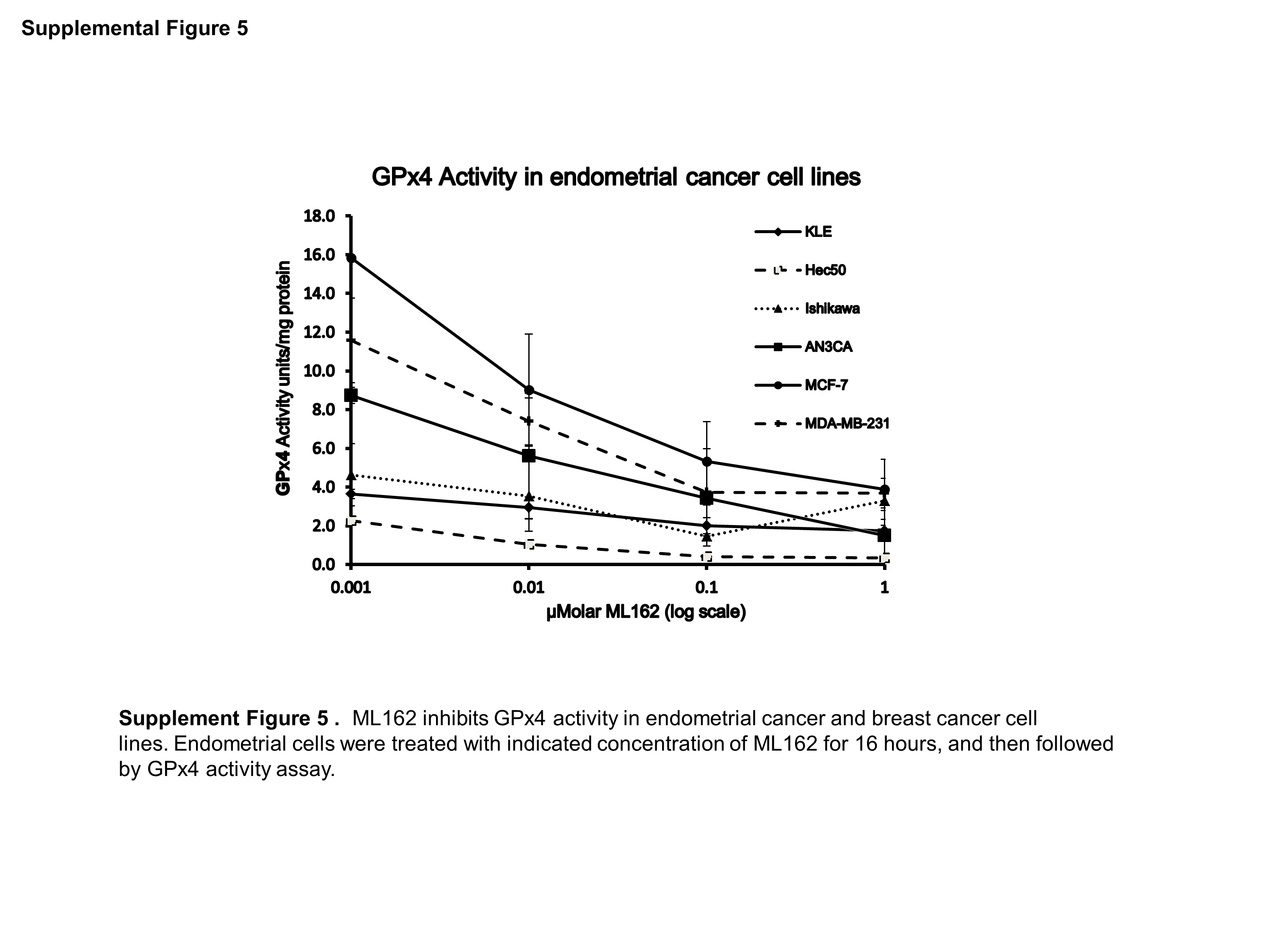

Supplement: Supplementary file 6 — Supplemental figure 5 [file 41419_2019_1897_MOESM6_ESM.tif]

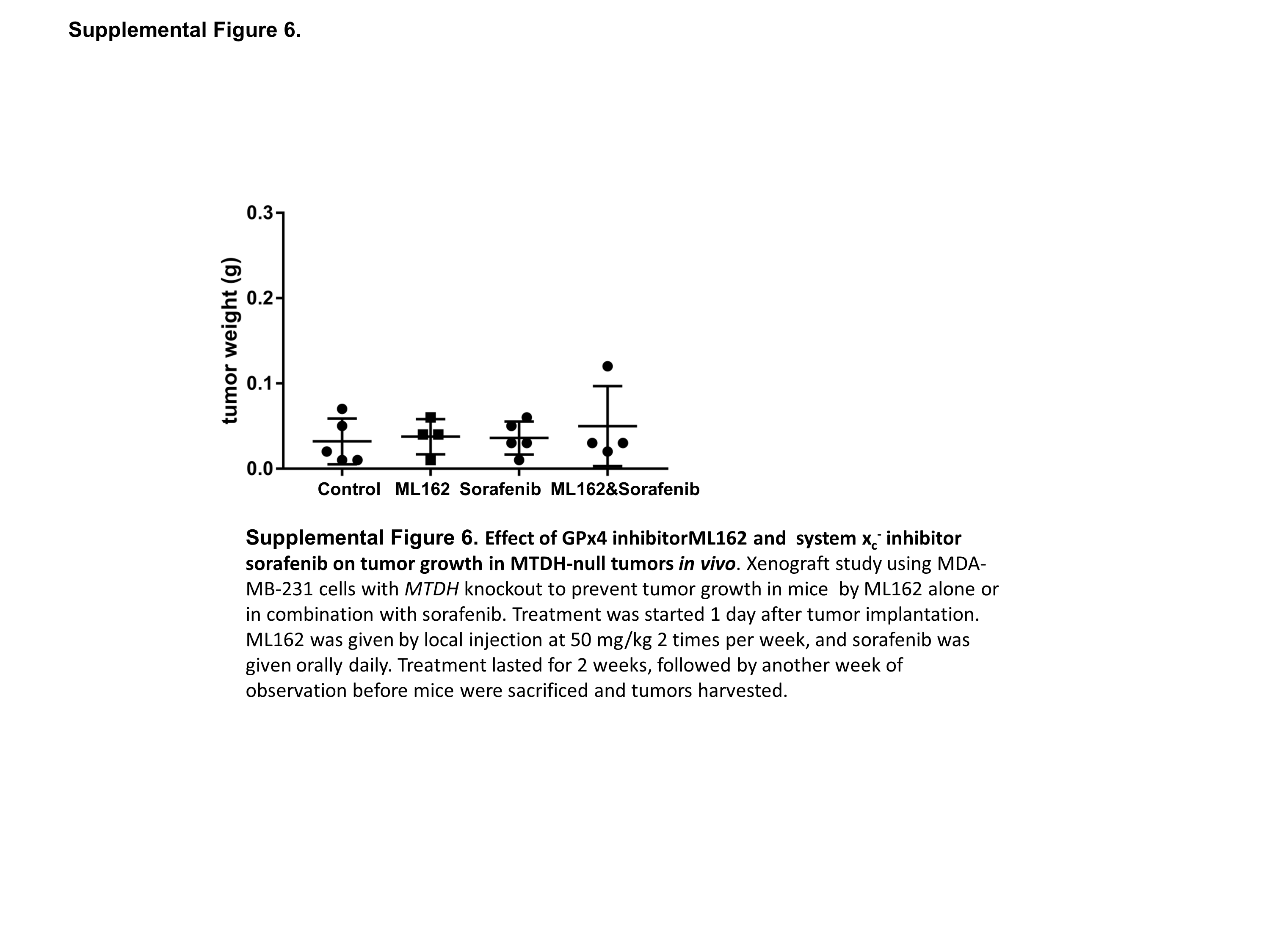

Supplement: Supplementary file 7 — Supplemental figure 6 [file 41419_2019_1897_MOESM7_ESM.tif]
