# Supplementary material for: Metadherin enhances vulnerability of cancer cells to ferroptosis
Source: Cell Death Dis. 2019 Sep 17;10(10):682. doi: 10.1038/s41419-019-1897-2 (PMC6746770; doi:10.1038/s41419-019-1897-2)
Supplement: Supplementary file 8 — Supplemental table 1 [file 41419_2019_1897_MOESM8_ESM.docx]

**Supplemental Table 1.** Correlating E-cadherin/β-actin ratio with ML162 IC50s in 11 cancer cell lines. The E-cadherin/β-actin ratio was determined using Western blot followed by densitometry. In general, low E-cadherin/β-actin ratio (i.e. mesenchymal-high) correlated with increased sensitivity to GPX4 inhibitor ML162.

| **Cell line** | **Cancer type** | **E-cadherin/β-actin ratio** | **ML162 IC50 (mM)** |
| --- | --- | --- | --- |
| **Considered as sensitive to ML162 (IC50 <= 1mM)** | | | |
| DMS53 | SCLC | 0.467843939 | S(0.5) |
| DMS273 | SCLC | 0.001670238 | S(0.7) |
| MDA-MB-231 | Breast | 0.01 | S(0.5) |
| KLE | Endometrial | 0.02 | S(0.4) |
| AN3CA | Endometrial | 0.006 | S(0.6) |
| RL95 | Endometrial | 0.07 | S(0.5) |
| Hec50 | Endometrial | 0.004 | S(0.4) |
| **Considered as resistant to ML162 (IC50 > 1mM)** | | | |
| A549 | NSCLC | 1.109250968 | R(8.5) |
| MCF7 | Breast | 1.22 | R(18) |
| Ishikawa | Endometrial | 0.877 | R(9) |
| Hec1A | Endometrial | 0.61 | R(6.8) |
