# Supplementary material for: Metadherin enhances vulnerability of cancer cells to ferroptosis
Source: Cell Death Dis. 2019 Sep 17;10(10):682. doi: 10.1038/s41419-019-1897-2 (PMC6746770; doi:10.1038/s41419-019-1897-2)
Supplement: Supplementary file 9 — Supplemental table 2 [file 41419_2019_1897_MOESM9_ESM.docx]

**Supplemental Table 2.** Enrichment of sequences of mRNAs encoding for SLC3A2, SLC7A11 and GPX4 by PAR-CLIP of MTDH antibody. 5’UTR: 5’ untranslated region. CDS: coding sequence. 3’UTR: 3’ untranslated region. Derived from GSE110260 in HepG2 cells stored at GEO.

| **#Refseq** | **Gene** | **Cluster start** | **Cluster end** | **Cluster score** |  |
| --- | --- | --- | --- | --- | --- |
| NM_001012664 | SLC3A2 | 144 | 245 | 8.583663 | \| **5’UTR** \| \| --- \| |
| NM_001012664 | SLC3A2 | 248 | 597 | 9.168626 |  |
| NM_001012664 | SLC3A2 | 607 | 668 | 7.583663 | \| **CDS** \| \| --- \| |
| NM_001012664 | SLC3A2 | 722 | 863 | 12.33855 |  |
| NM_001012664 | SLC3A2 | 1872 | 2091 | 9.776308 | **3’UTR** |
|  |  |  |  |  |  |
| NM_014331 | SLC7A11 | 687 | 783 | 9.749259 | **CDS** |
| NM_014331 | SLC7A11 | 1189 | 1219 | 7.427324 |  |
| NM_014331 | SLC7A11 | 1238 | 1299 | 8.427331 |  |
| NM_014331 | SLC7A11 | 1238 | 1299 | 8.427331 |  |
| NM_014331 | SLC7A11 | 1367 | 1497 | 9.749259 |  |
| NM_014331 | SLC7A11 | 1600 | 1668 | 9.749259 |  |
| NM_014331 | SLC7A11 | 1763 | 1834 | 9.012287 | **3’UTR** |
| NM_014331 | SLC7A11 | 7535 | 7643 | 7.423314 |  |
| NM_014331 | SLC7A11 | 8398 | 8475 | 7.343192 |  |
| NM_014331 | SLC7A11 | 8755 | 8827 | 8.427331 |  |
|  |  |  |  |  |  |
| NM_001039848 | GPX4 | 284 | 396 | 6.749819 | **CDS** |
| NM_001039848 | GPX4 | 418 | 589 | 6.901822 |  |
| NM_001039848 | GPX4 | 672 | 727 | 3.579894 |  |
| NM_001039848 | GPX4 | 727 | 787 | 3.57972 |  |
| NM_001039848 | GPX4 | 836 | 893 | 3.495755 | **3’UTR** |
