# Supplementary material for: Metadherin enhances vulnerability of cancer cells to ferroptosis
Source: Cell Death Dis. 2019 Sep 17;10(10):682. doi: 10.1038/s41419-019-1897-2 (PMC6746770; doi:10.1038/s41419-019-1897-2)
Supplement: Supplementary file 10 — Supplemental table 3 [file 41419_2019_1897_MOESM10_ESM.docx]

| **Supplemental Table 3.** MTDH induces metabolomic changes that increase the susceptibility to ferroptosis. | | | | | | | | |  |  |  |
| --- | --- | --- | --- | --- | --- | --- | --- | --- | --- | --- | --- |
|  | A comprehensive metabolomics study was performed using isogenic MDA-MB-231 cells with alternative MTDH (WT vs. KO). | | | | | | | | | |  |
| **sample** | **class** | **2- Hydroxyglutarate** | **3- Phosphoglycerate** | **6-Phosphogluconate** | **Aconitate** | **Alanine** | **alpha.Keto.beta.Methylvalerate..KMV.** | **alpha.Ketoglutarate** | **alpha.Ketoisocaproate..KIC.** | **Aminoadipate** |  |
| Sample 1 (Control 1) | Control | 1140.4 | 1725.7 | 829.4 | 1259.2 | 929.8 | 1165.3 | 1247.4 | 1739.0 | 922.2 |  |
| Sample 2 (Control 2) | Control | 1200.2 | 1728.9 | 1034.1 | 1415.4 | 1003.2 | 1327.5 | 1494.7 | 2308.3 | 1028.1 |  |
| Sample 3 (Control 3) | Control | 1118.4 | 1643.9 | 981.4 | 1381.5 | 1022.1 | 1232.5 | 1305.9 | 1798.2 | 1006.3 |  |
| Sample 4 (Control 4) | Control | 1296.1 | 1716.0 | 1222.4 | 1642.9 | 1021.2 | 1375.7 | 1243.4 | 1637.5 | 1012.1 |  |
| Sample 5 (Control 5) | Control | 1099.6 | 1508.7 | 1285.7 | 1417.6 | 1016.4 | 1290.4 | 1325.6 | 989.3 | 962.2 |  |
| Sample 6 (Control 6) | Control | 1310.5 | 1616.0 | 1453.5 | 1653.7 | 1025.1 | 1235.4 | 1161.3 | 973.5 | 1112.4 |  |
|  |  |  |  |  |  |  |  |  |  |  |  |
|  | **Mean** | 1194.2 | 1656.5 | 1134.4 | 1461.7 | 1003.0 | 1271.1 | 1296.4 | 1574.3 | 1007.2 |  |
|  | **SE** | 37.2 | 35.2 | 92.9 | 63.6 | 15.0 | 30.8 | 46.1 | 210.0 | 26.3 |  |
|  | **CV** | 8% | 5% | 20% | 11% | 4% | 6% | 9% | 33% | 6% |  |
|  |  |  |  |  |  |  |  |  |  |  |  |
| Sample 7 (MKO-1) | MKO | 563.7 | 473.9 | 584.0 | 568.8 | 901.9 | 631.7 | 450.5 | 999.2 | 741.8 |  |
| Sample 8 (MKO-2) | MKO | 646.8 | 564.8 | 684.5 | 702.4 | 970.0 | 740.1 | 541.3 | 974.5 | 878.6 |  |
| Sample 9 (MKO-3) | MKO | 684.3 | 682.5 | 736.8 | 734.8 | 1029.6 | 795.5 | 594.2 | 929.6 | 909.8 |  |
| Sample 10 (MKO-4) | MKO | 688.8 | 515.9 | 785.7 | 763.8 | 1060.0 | 732.5 | 586.1 | 600.3 | 900.8 |  |
| Sample 11 (MKO-5) | MKO | 763.4 | 693.5 | 1032.7 | 874.5 | 1118.2 | 960.1 | 683.5 | 681.7 | 1084.3 |  |
| Sample 12 (MKO-6) | MKO | 851.4 | 657.1 | 1180.0 | 948.6 | 1320.1 | 959.9 | 791.1 | 623.8 | 1072.5 |  |
|  |  |  |  |  |  |  |  |  |  |  |  |
|  | **Mean** | 699.7 | 598.0 | 833.9 | 765.5 | 1066.6 | 803.3 | 607.8 | 801.5 | 931.3 |  |
|  | **SE** | 40.3 | 37.9 | 92.3 | 54.5 | 59.1 | 54.0 | 48.0 | 75.7 | 52.7 |  |
|  | **CV** | 14% | 16% | 27% | 17% | 14% | 16% | 19% | 23% | 14% |  |
|  |  |  |  |  |  |  |  |  |  |  |  |
|  | **t-test** |  |  |  |  |  |  |  |  |  |  |
|  |  | **2- Hydroxyglutarate** | **3- Phosphoglycerate** | **6-Phosphogluconate** | **Aconitate** | **Alanine** | **alpha.Keto.beta.Methylvalerate..KMV.** | **alpha.Ketoglutarate** | **alpha.Ketoisocaproate..KIC.** | **Aminoadipate** |  |
|  | **MKO/Control** | 0.5860 | 0.3610 | 0.7351 | 0.5237 | 1.0635 | 0.6320 | 0.4688 | 0.5091 | 0.9246 |  |
|  | **P-value** | 0.0000 | 0.0000 | 0.0447 | 0.0000 | 0.3210 | 0.0000 | 0.0000 | 0.0061 | 0.2267 |  |
|  |  |  |  |  |  |  |  |  |  |  |  |

| **sample** | **class** | **Arachidic.acid..Eicosanoic.acid.** | **Arachidonate** | **Asparagine** | **Aspartate** | **Behenic.acid..Docosanoic.acid.** | **beta.Alanine** | **beta.Hydroxybutyrate..3.Hydroxybutyrate.** | **Cholesterol** | **Citrate** |
| --- | --- | --- | --- | --- | --- | --- | --- | --- | --- | --- |
| Sample 1 (Control 1) | Control | 1002.6 | 1757.9 | 893.0 | 1132.1 | 1064.6 | 1167.6 | 1092.7 | 1235.7 | 1272.9 |
| Sample 2 (Control 2) | Control | 900.7 | 782.7 | 861.6 | 1244.0 | 968.4 | 1207.4 | 1109.7 | 1394.7 | 1459.0 |
| Sample 3 (Control 3) | Control | 830.2 | 1100.3 | 808.9 | 1233.8 | 854.0 | 1191.9 | 940.0 | 1346.1 | 1396.7 |
| Sample 4 (Control 4) | Control | 904.9 | 938.9 | 829.8 | 1242.9 | 509.7 | 1299.5 | 1032.3 | 1471.0 | 1464.0 |
| Sample 5 (Control 5) | Control | 886.0 | 1110.0 | 879.1 | 1212.4 | 878.3 | 1257.6 | 813.8 | 1352.8 | 1333.4 |
| Sample 6 (Control 6) | Control | 897.7 | 836.9 | 796.1 | 1239.6 | 935.0 | 1308.5 | 959.2 | 1477.3 | 1486.8 |
|  |  |  |  |  |  |  |  |  |  |  |
|  | **Mean** | 903.7 | 1087.8 | 844.8 | 1217.4 | 868.3 | 1238.8 | 991.3 | 1379.6 | 1402.1 |
|  | **SE** | 22.8 | 144.7 | 16.0 | 17.7 | 77.9 | 23.9 | 45.1 | 36.8 | 34.5 |
|  | **CV** | 6% | 33% | 5% | 4% | 22% | 5% | 11% | 7% | 6% |
|  |  |  |  |  |  |  |  |  |  |  |
| Sample 7 (MKO-1) | MKO | 846.7 | 1332.8 | 877.8 | 754.0 | 729.8 | 656.3 | 713.7 | 588.4 | 569.2 |
| Sample 8 (MKO-2) | MKO | 813.5 | 1336.8 | 891.1 | 758.1 | 729.1 | 709.9 | 725.4 | 689.8 | 629.2 |
| Sample 9 (MKO-3) | MKO | 875.8 | 1449.4 | 1121.4 | 855.0 | 711.8 | 690.6 | 716.4 | 722.4 | 680.2 |
| Sample 10 (MKO-4) | MKO | 816.0 | 1312.8 | 865.1 | 825.7 | 643.2 | 686.5 | 701.7 | 679.9 | 632.4 |
| Sample 11 (MKO-5) | MKO | 815.5 | 1246.1 | 994.5 | 861.6 | 758.7 | 718.6 | 848.3 | 760.7 | 713.4 |
| Sample 12 (MKO-6) | MKO | 1003.2 | 1130.2 | 1146.5 | 977.6 | 963.6 | 791.6 | 869.9 | 825.2 | 815.3 |
|  |  |  |  |  |  |  |  |  |  |  |
|  | **Mean** | 861.8 | 1301.4 | 982.8 | 838.7 | 756.0 | 708.9 | 762.6 | 711.1 | 673.3 |
|  | **SE** | 30.0 | 43.5 | 51.5 | 33.6 | 44.4 | 18.7 | 30.8 | 32.7 | 34.8 |
|  | **CV** | 9% | 8% | 13% | 10% | 14% | 6% | 10% | 11% | 13% |
|  |  |  |  |  |  |  |  |  |  |  |
|  | **t-test** |  |  |  |  |  |  |  |  |  |
|  |  | **Arachidic.acid..Eicosanoic.acid.** | **Arachidonate** | **Asparagine** | **Aspartate** | **Behenic.acid..Docosanoic.acid.** | **beta.Alanine** | **beta.Hydroxybutyrate..3.Hydroxybutyrate.** | **Cholesterol** | **Citrate** |
|  | **MKO/Control** | 0.9537 | 1.1963 | 1.1634 | 0.6889 | 0.8707 | 0.5723 | 0.7693 | 0.5154 | 0.4802 |
|  | **P-value** | 0.2922 | 0.1878 | 0.0283 | 0.0000 | 0.2389 | 0.0000 | 0.0019 | 0.0000 | 0.0000 |

| **sample** | **class** | **Citrulline** | **Cysteine** | **Dihydroxyacetone.phosphate..DHAP.** | **Fructose** | **Fructose.6.phosphate** | **Fumarate** | **Gamma.aminobutyric.acid..GABA.** | Glucose | **Glucose.6.phosphate** |
| --- | --- | --- | --- | --- | --- | --- | --- | --- | --- | --- |
| Sample 1 (Control 1) | Control | 858.7 | 583.6 | 712.5 | 1275.5 | 800.6 | 1188.8 | 1304.4 | 1782.8 | 880.5 |
| Sample 2 (Control 2) | Control | 915.0 | 632.2 | 790.7 | 1382.4 | 1036.4 | 1354.1 | 1456.4 | 1678.4 | 1100.1 |
| Sample 3 (Control 3) | Control | 844.5 | 611.5 | 776.7 | 1277.9 | 1093.9 | 1311.8 | 1440.9 | 1435.0 | 1069.9 |
| Sample 4 (Control 4) | Control | 702.5 | 614.3 | 831.5 | 1396.5 | 1279.4 | 1420.6 | 1473.9 | 1299.9 | 1430.3 |
| Sample 5 (Control 5) | Control | 700.6 | 595.1 | 962.0 | 1254.7 | 1201.9 | 1303.1 | 1397.4 | 1232.1 | 1351.6 |
| Sample 6 (Control 6) | Control | 814.1 | 572.3 | 1051.1 | 1316.7 | 1301.4 | 1393.3 | 1383.0 | 1227.5 | 1380.1 |
|  |  |  |  |  |  |  |  |  |  |  |
|  | **Mean** | 805.9 | 601.5 | 854.1 | 1317.3 | 1118.9 | 1328.6 | 1409.3 | 1442.6 | 1202.1 |
|  | **SE** | 35.6 | 9.0 | 52.0 | 24.3 | 76.3 | 33.6 | 25.3 | 97.0 | 89.0 |
|  | **CV** | 11% | 4% | 15% | 5% | 17% | 6% | 4% | 16% | 18% |
|  |  |  |  |  |  |  |  |  |  |  |
| Sample 7 (MKO-1) | MKO | 917.9 | 1150.4 | 837.7 | 687.7 | 618.9 | 612.7 | 597.5 | 654.4 | 298.0 |
| Sample 8 (MKO-2) | MKO | 897.7 | 1283.3 | 873.4 | 756.2 | 673.7 | 662.5 | 632.0 | 648.7 | 786.8 |
| Sample 9 (MKO-3) | MKO | 847.1 | 1483.1 | 961.7 | 771.8 | 753.3 | 713.5 | 675.5 | 539.6 | 861.0 |
| Sample 10 (MKO-4) | MKO | 878.9 | 1382.9 | 996.8 | 726.1 | 829.8 | 728.0 | 659.8 | 330.7 | 859.9 |
| Sample 11 (MKO-5) | MKO | 850.6 | 1532.5 | 1235.1 | 816.0 | 917.6 | 778.3 | 667.8 | 418.7 | 1043.4 |
| Sample 12 (MKO-6) | MKO | 1503.1 | 1746.2 | 1299.2 | 974.0 | 1120.6 | 864.6 | 716.1 | 594.0 | 1111.8 |
|  |  |  |  |  |  |  |  |  |  |  |
|  | **Mean** | 982.5 | 1429.7 | 1034.0 | 788.6 | 819.0 | 726.6 | 658.1 | 531.0 | 826.8 |
|  | **SE** | 104.7 | 84.7 | 77.8 | 41.0 | 74.4 | 36.0 | 16.4 | 53.5 | 117.1 |
|  | **CV** | 26% | 15% | 18% | 13% | 22% | 12% | 6% | 25% | 35% |
|  |  |  |  |  |  |  |  |  |  |  |
|  | **t-test** |  |  |  |  |  |  |  |  |  |
|  |  | **Citrulline** | **Cysteine** | **Dihydroxyacetone.phosphate..DHAP.** | **Fructose** | **Fructose.6.phosphate** | **Fumarate** | **Gamma.aminobutyric.acid..GABA.** | **Glucose** | **Glucose.6.phosphate** |
|  | **MKO/Control** | 1.2192 | 2.3770 | 1.2106 | 0.5987 | 0.7320 | 0.5469 | 0.4670 | 0.3681 | 0.6878 |
|  | **P-value** | 0.1413 | 0.0000 | 0.0835 | 0.0000 | 0.0184 | 0.0000 | 0.0000 | 0.0000 | 0.0288 |

| **sample** | **class** | **Glutamate** | **Glutamine** | **Glycerate** | **Glycerol.Monolaurate..GML.** | **Glycine** | **Guanine** | **Heptadecanoic.acid** | **Histidine** | **Hypotaurine** |
| --- | --- | --- | --- | --- | --- | --- | --- | --- | --- | --- |
| Sample 1 (Control 1) | Control | 1066.0 | 1018.5 | 1536.4 | 1010.8 | 607.5 | 1363.7 | 1087.1 | 759.8 | 1514.5 |
| Sample 2 (Control 2) | Control | 1146.8 | 1040.1 | 1402.0 | 1121.3 | 660.4 | 1286.7 | 982.4 | 895.6 | 1418.1 |
| Sample 3 (Control 3) | Control | 1116.5 | 1041.0 | 1154.0 | 1120.7 | 634.9 | 1207.2 | 897.4 | 834.6 | 1427.8 |
| Sample 4 (Control 4) | Control | 1168.4 | 1126.0 | 1218.4 | 1171.6 | 668.8 | 1597.1 | 953.7 | 885.7 | 1530.4 |
| Sample 5 (Control 5) | Control | 1108.7 | 1048.6 | 1327.9 | 1057.4 | 961.1 | 1191.8 | 1004.9 | 913.7 | 1388.7 |
| Sample 6 (Control 6) | Control | 1161.1 | 1035.1 | 1047.0 | 883.4 | 679.9 | 1214.4 | 951.9 | 945.0 | 1250.8 |
|  |  |  |  |  |  |  |  |  |  |  |
|  | **Mean** | 1127.9 | 1051.6 | 1281.0 | 1060.9 | 702.1 | 1310.1 | 979.6 | 872.4 | 1421.7 |
|  | **SE** | 15.7 | 15.4 | 72.3 | 42.2 | 52.9 | 63.1 | 26.1 | 27.0 | 41.2 |
|  | **CV** | 3% | 4% | 14% | 10% | 18% | 12% | 7% | 8% | 7% |
|  |  |  |  |  |  |  |  |  |  |  |
| Sample 7 (MKO-1) | MKO | 760.4 | 944.7 | 845.5 | 1068.1 | 1164.3 | 1008.3 | 805.6 | 688.5 | 602.4 |
| Sample 8 (MKO-2) | MKO | 825.3 | 978.4 | 933.1 | 1121.8 | 738.5 | 1417.7 | 797.2 | 787.4 | 690.0 |
| Sample 9 (MKO-3) | MKO | 873.3 | 1063.4 | 882.3 | 1058.1 | 691.2 | 1027.1 | 820.6 | 894.9 | 684.8 |
| Sample 10 (MKO-4) | MKO | 854.7 | 994.2 | 1059.8 | 960.5 | 3197.0 | 1118.2 | 834.7 | 835.4 | 638.8 |
| Sample 11 (MKO-5) | MKO | 922.3 | 1038.2 | 945.3 | 1344.4 | 715.7 | 1179.3 | 839.4 | 963.0 | 678.9 |
| Sample 12 (MKO-6) | MKO | 1017.5 | 1194.6 | 1077.3 | 1030.4 | 1037.4 | 1081.1 | 1164.4 | 1504.4 | 676.2 |
|  |  |  |  |  |  |  |  |  |  |  |
|  | **Mean** | 875.6 | 1035.6 | 957.2 | 1097.2 | 1257.3 | 1138.6 | 877.0 | 945.6 | 661.9 |
|  | **SE** | 35.9 | 36.2 | 38.2 | 53.9 | 395.9 | 61.3 | 57.9 | 118.1 | 14.0 |
|  | **CV** | 10% | 9% | 10% | 12% | 77% | 13% | 16% | 31% | 5% |
|  |  |  |  |  |  |  |  |  |  |  |
|  | **t-test** |  |  |  |  |  |  |  |  |  |
|  |  | **Glutamate** | **Glutamine** | **Glycerate** | **Glycerol.Monolaurate..GML.** | **Glycine** | **Guanine** | **Heptadecanoic.acid** | **Histidine** | **Hypotaurine** |
|  | **MKO/Control** | 0.7763 | 0.9848 | 0.7473 | 1.0343 | 1.7908 | 0.8691 | 0.8953 | 1.0839 | 0.4655 |
|  | **P-value** | 0.0001 | 0.6934 | 0.0027 | 0.6073 | 0.1947 | 0.0798 | 0.1371 | 0.5590 | 0.0000 |

| **sample** | **class** | **Inotisol** | **Isocitrate** | **Isoleucine** | **Lactate** | **Lauric.acid** | **Leucine** | **Linoleate** | **Lysine** | **Malate** |
| --- | --- | --- | --- | --- | --- | --- | --- | --- | --- | --- |
| Sample 1 (Control 1) | Control | 1481.1 | 1199.8 | 898.8 | 1078.9 | 1117.6 | 906.8 | 1159.4 | 929.2 | 1233.7 |
| Sample 2 (Control 2) | Control | 1629.0 | 1267.1 | 1018.0 | 1186.5 | 1153.4 | 1035.8 | 1294.8 | 974.7 | 1397.5 |
| Sample 3 (Control 3) | Control | 1578.7 | 1321.1 | 1022.3 | 1118.4 | 1151.5 | 1063.0 | 1328.0 | 1031.3 | 1333.1 |
| Sample 4 (Control 4) | Control | 1687.3 | 1431.8 | 924.2 | 1254.0 | 1127.9 | 953.1 | 1349.5 | 991.6 | 1394.1 |
| Sample 5 (Control 5) | Control | 1566.6 | 1265.0 | 966.0 | 1154.9 | 1124.9 | 1010.7 | 1373.8 | 960.4 | 1286.2 |
| Sample 6 (Control 6) | Control | 1648.5 | 1450.9 | 992.1 | 1190.2 | 1145.4 | 1041.1 | 1329.4 | 1001.6 | 1387.6 |
|  |  |  |  |  |  |  |  |  |  |  |
|  | **Mean** | 1598.5 | 1322.6 | 970.2 | 1163.8 | 1136.8 | 1001.7 | 1305.8 | 981.5 | 1338.7 |
|  | **SE** | 29.7 | 40.8 | 20.6 | 25.0 | 6.2 | 24.4 | 31.2 | 14.4 | 27.5 |
|  | **CV** | 5% | 8% | 5% | 5% | 1% | 6% | 6% | 4% | 5% |
|  |  |  |  |  |  |  |  |  |  |  |
| Sample 7 (MKO-1) | MKO | 354.5 | 478.8 | 962.8 | 708.7 | 923.3 | 949.2 | 914.6 | 1016.1 | 596.9 |
| Sample 8 (MKO-2) | MKO | 387.7 | 521.8 | 1005.0 | 800.2 | 924.8 | 1002.1 | 1046.1 | 1012.4 | 649.6 |
| Sample 9 (MKO-3) | MKO | 408.8 | 566.5 | 1143.3 | 843.5 | 844.7 | 1160.9 | 1138.2 | 1018.8 | 686.0 |
| Sample 10 (MKO-4) | MKO | 396.7 | 641.0 | 1111.6 | 828.2 | 909.9 | 1114.9 | 1130.0 | 995.1 | 681.5 |
| Sample 11 (MKO-5) | MKO | 438.9 | 708.4 | 1080.6 | 916.4 | 912.8 | 1069.3 | 1107.9 | 1050.0 | 747.7 |
| Sample 12 (MKO-6) | MKO | 469.8 | 934.9 | 1308.7 | 1016.4 | 918.0 | 1327.6 | 1069.9 | 1127.9 | 853.5 |
|  |  |  |  |  |  |  |  |  |  |  |
|  | **Mean** | 409.4 | 641.9 | 1102.0 | 852.2 | 905.6 | 1104.0 | 1067.8 | 1036.7 | 702.5 |
|  | **SE** | 16.5 | 67.6 | 49.6 | 42.8 | 12.4 | 54.4 | 33.8 | 19.6 | 36.3 |
|  | **CV** | 10% | 26% | 11% | 12% | 3% | 12% | 8% | 5% | 13% |
|  |  |  |  |  |  |  |  |  |  |  |
|  | **t-test** |  |  |  |  |  |  |  |  |  |
|  |  | **Inotisol** | **Isocitrate** | **Isoleucine** | **Lactate** | **Lauric.acid** | **Leucine** | **Linoleate** | **Lysine** | **Malate** |
|  | **MKO/Control** | 0.2561 | 0.4853 | 1.1358 | 0.7323 | 0.7966 | 1.1021 | 0.8177 | 1.0563 | 0.5248 |
|  | **P-value** | 0.0000 | 0.0000 | 0.0340 | 0.0001 | 0.0000 | 0.1174 | 0.0004 | 0.0466 | 0.0000 |

| **sample** | **class** | **Methionine** | **Myristic.acid** | **N.Acetylaspartate** | **N.Acetylglutamate** | **N.Acetylserine** | **Oleic.acid** | **O.Phosphoethanolamine** | **Ornithine** | **Orotate** |
| --- | --- | --- | --- | --- | --- | --- | --- | --- | --- | --- |
| Sample 1 (Control 1) | Control | 888.6 | 1025.0 | 1295.8 | 1171.4 | 758.8 | 998.0 | 1337.7 | 787.4 | 931.4 |
| Sample 2 (Control 2) | Control | 957.7 | 1010.5 | 1575.8 | 1400.3 | 1187.9 | 914.1 | 1160.1 | 747.2 | 1060.1 |
| Sample 3 (Control 3) | Control | 999.2 | 943.8 | 1491.3 | 1310.6 | 1153.7 | 890.8 | 1064.8 | 764.3 | 1045.8 |
| Sample 4 (Control 4) | Control | 1016.5 | 1034.7 | 1419.6 | 1234.2 | 764.3 | 892.6 | 1126.6 | 715.7 | 1058.7 |
| Sample 5 (Control 5) | Control | 989.4 | 1013.7 | 1398.9 | 1224.1 | 1194.1 | 952.7 | 1045.6 | 999.4 | 972.7 |
| Sample 6 (Control 6) | Control | 978.8 | 1028.1 | 1549.3 | 1405.8 | 1168.8 | 988.4 | 1067.4 | 674.1 | 1114.9 |
|  |  |  |  |  |  |  |  |  |  |  |
|  | **Mean** | 971.7 | 1009.3 | 1455.1 | 1291.1 | 1037.9 | 939.4 | 1133.7 | 781.4 | 1030.6 |
|  | **SE** | 18.5 | 13.6 | 42.6 | 39.8 | 87.6 | 19.3 | 44.4 | 46.5 | 27.2 |
|  | **CV** | 5% | 3% | 7% | 8% | 21% | 5% | 10% | 15% | 6% |
|  |  |  |  |  |  |  |  |  |  |  |
| Sample 7 (MKO-1) | MKO | 962.9 | 837.5 | 518.6 | 531.8 | 693.9 | 745.1 | 975.5 | 1020.3 | 741.8 |
| Sample 8 (MKO-2) | MKO | 1030.6 | 914.6 | 576.9 | 565.5 | 728.2 | 783.8 | 1017.1 | 1012.2 | 793.0 |
| Sample 9 (MKO-3) | MKO | 1169.0 | 891.0 | 628.8 | 652.6 | 860.3 | 756.4 | 993.9 | 975.9 | 880.8 |
| Sample 10 (MKO-4) | MKO | 1161.3 | 909.8 | 593.9 | 614.0 | 1210.9 | 766.4 | 776.3 | 940.7 | 773.7 |
| Sample 11 (MKO-5) | MKO | 1207.6 | 949.8 | 638.6 | 649.3 | 770.8 | 860.3 | 876.7 | 994.4 | 950.7 |
| Sample 12 (MKO-6) | MKO | 1333.2 | 1242.7 | 782.3 | 779.6 | 1429.5 | 997.2 | 933.4 | 3274.1 | 1106.3 |
|  |  |  |  |  |  |  |  |  |  |  |
|  | **Mean** | 1144.1 | 957.6 | 623.2 | 632.2 | 948.9 | 818.2 | 928.8 | 1369.6 | 874.4 |
|  | **SE** | 53.7 | 59.0 | 36.3 | 35.3 | 122.9 | 39.5 | 36.6 | 381.1 | 56.0 |
|  | **CV** | 11% | 15% | 14% | 14% | 32% | 12% | 10% | 68% | 16% |
|  |  |  |  |  |  |  |  |  |  |  |
|  | **t-test** |  |  |  |  |  |  |  |  |  |
|  |  | **Methionine** | **Myristic.acid** | **N.Acetylaspartate** | **N.Acetylglutamate** | **N.Acetylserine** | **Oleic.acid** | **O.Phosphoethanolamine** | **Ornithine** | **Orotate** |
|  | **MKO/Control** | 1.1774 | 0.9487 | 0.4283 | 0.4896 | 0.9142 | 0.8709 | 0.8193 | 1.7529 | 0.8484 |
|  | **P-value** | 0.0125 | 0.4126 | 0.0000 | 0.0000 | 0.5684 | 0.0203 | 0.0052 | 0.1564 | 0.0309 |

| **sample** | **class** | **Palmitate** | **Phenylalanine** | **Phosphoenolpyruvate** | **Proline** | **Pyruvate** | **Ribose.5.phosphate** | **Serine** | **Stearate** | **Succinate** |
| --- | --- | --- | --- | --- | --- | --- | --- | --- | --- | --- |
| Sample 1 (Control 1) | Control | 874.7 | 870.4 | 1017.1 | 974.1 | 1321.1 | 951.4 | 964.6 | 935.8 | 1510.3 |
| Sample 2 (Control 2) | Control | 837.9 | 946.1 | 1045.1 | 1048.3 | 1498.0 | 971.6 | 999.2 | 898.4 | 1523.8 |
| Sample 3 (Control 3) | Control | 791.7 | 933.0 | 983.5 | 1149.8 | 1352.5 | 893.5 | 1061.0 | 810.1 | 1365.2 |
| Sample 4 (Control 4) | Control | 912.0 | 932.7 | 963.1 | 990.4 | 1122.0 | 953.3 | 1001.8 | 927.3 | 1653.3 |
| Sample 5 (Control 5) | Control | 877.9 | 913.6 | 890.9 | 1056.8 | 1457.5 | 908.1 | 995.2 | 896.7 | 1265.3 |
| Sample 6 (Control 6) | Control | 844.8 | 944.2 | 962.5 | 1153.0 | 902.4 | 1091.5 | 1013.5 | 886.4 | 1914.6 |
|  |  |  |  |  |  |  |  |  |  |  |
|  | **Mean** | 856.5 | 923.3 | 977.0 | 1062.1 | 1275.6 | 961.6 | 1005.9 | 892.5 | 1538.7 |
|  | **SE** | 16.9 | 11.6 | 21.7 | 31.1 | 91.9 | 28.7 | 12.9 | 18.2 | 93.2 |
|  | **CV** | 5% | 3% | 5% | 7% | 18% | 7% | 3% | 5% | 15% |
|  |  |  |  |  |  |  |  |  |  |  |
| Sample 7 (MKO-1) | MKO | 797.4 | 968.1 | 806.0 | 875.5 | 539.6 | 710.7 | 1003.7 | 863.4 | 691.7 |
| Sample 8 (MKO-2) | MKO | 806.4 | 998.8 | 889.5 | 931.7 | 650.6 | 764.4 | 1039.0 | 855.8 | 709.7 |
| Sample 9 (MKO-3) | MKO | 866.0 | 1105.8 | 805.7 | 1092.3 | 720.4 | 793.2 | 1185.2 | 903.5 | 718.3 |
| Sample 10 (MKO-4) | MKO | 802.4 | 1060.8 | 816.4 | 1114.7 | 662.3 | 867.3 | 1208.0 | 871.8 | 622.0 |
| Sample 11 (MKO-5) | MKO | 834.8 | 1157.2 | 839.5 | 1063.4 | 793.1 | 881.1 | 1204.0 | 893.3 | 713.1 |
| Sample 12 (MKO-6) | MKO | 882.7 | 1271.3 | 923.5 | 1340.8 | 912.6 | 1098.7 | 1420.9 | 918.9 | 797.0 |
|  |  |  |  |  |  |  |  |  |  |  |
|  | **Mean** | 831.6 | 1093.7 | 846.8 | 1069.7 | 713.1 | 852.6 | 1176.8 | 884.4 | 708.6 |
|  | **SE** | 14.7 | 45.3 | 20.0 | 66.5 | 52.5 | 55.7 | 60.6 | 10.1 | 22.9 |
|  | **CV** | 4% | 10% | 6% | 15% | 18% | 16% | 13% | 3% | 8% |
|  |  |  |  |  |  |  |  |  |  |  |
|  | **t-test** |  |  |  |  |  |  |  |  |  |
|  |  | **Palmitate** | **Phenylalanine** | **Phosphoenolpyruvate** | **Proline** | **Pyruvate** | **Ribose.5.phosphate** | **Serine** | **Stearate** | **Succinate** |
|  | **MKO/Control** | 0.9710 | 1.1845 | 0.8667 | 1.0072 | 0.5590 | 0.8866 | 1.1700 | 0.9910 | 0.4605 |
|  | **P-value** | 0.2923 | 0.0045 | 0.0013 | 0.9190 | 0.0003 | 0.1124 | 0.0202 | 0.7079 | 0.0000 |

| **sample** | **class** | **Taurine** | **Threonine** | **Tryptophan** | **Tyrosine** | **Uracil** | **Urea** | **Valine** |
| --- | --- | --- | --- | --- | --- | --- | --- | --- |
| Sample 1 (Control 1) | Control | 1604.6 | 1024.9 | 871.3 | 860.7 | 1445.6 | 1069.3 | 853.0 |
| Sample 2 (Control 2) | Control | 1678.7 | 1034.9 | 948.2 | 924.5 | 1029.0 | 1197.1 | 963.9 |
| Sample 3 (Control 3) | Control | 1622.1 | 1084.4 | 926.4 | 912.2 | 890.4 | 1096.4 | 962.7 |
| Sample 4 (Control 4) | Control | 1731.3 | 1047.9 | 938.2 | 878.3 | 727.2 | 1100.1 | 939.3 |
| Sample 5 (Control 5) | Control | 1533.1 | 1015.3 | 916.3 | 835.8 | 714.4 | 1092.6 | 933.1 |
| Sample 6 (Control 6) | Control | 1868.9 | 1049.5 | 987.0 | 913.1 | 793.6 | 1123.7 | 962.9 |
|  |  |  |  |  |  |  |  |  |
|  | **Mean** | 1673.1 | 1042.8 | 931.2 | 887.4 | 933.4 | 1113.2 | 935.8 |
|  | **SE** | 47.8 | 9.9 | 15.6 | 14.3 | 113.0 | 18.2 | 17.4 |
|  | **CV** | 7% | 2% | 4% | 4% | 30% | 4% | 5% |
|  |  |  |  |  |  |  |  |  |
| Sample 7 (MKO-1) | MKO | 423.4 | 964.3 | 860.6 | 963.9 | 1144.8 | 830.9 | 928.7 |
| Sample 8 (MKO-2) | MKO | 465.1 | 1025.3 | 959.9 | 1010.1 | 921.5 | 834.9 | 999.3 |
| Sample 9 (MKO-3) | MKO | 468.7 | 1144.2 | 1063.4 | 1072.8 | 696.0 | 819.9 | 1104.6 |
| Sample 10 (MKO-4) | MKO | 459.7 | 1182.3 | 982.8 | 1008.4 | 824.5 | 847.3 | 1039.1 |
| Sample 11 (MKO-5) | MKO | 579.4 | 1172.7 | 1104.1 | 1102.2 | 692.2 | 871.0 | 1085.5 |
| Sample 12 (MKO-6) | MKO | 566.8 | 1309.7 | 1319.1 | 1266.3 | 731.7 | 1752.0 | 1291.8 |
|  |  |  |  |  |  |  |  |  |
|  | **Mean** | 493.9 | 1133.1 | 1048.3 | 1070.6 | 835.1 | 992.7 | 1074.9 |
|  | **SE** | 26.0 | 50.1 | 64.3 | 44.1 | 71.6 | 152.0 | 50.5 |
|  | **CV** | 13% | 11% | 15% | 10% | 21% | 38% | 12% |
|  |  |  |  |  |  |  |  |  |
|  | **t-test** |  |  |  |  |  |  |  |
|  |  | **Taurine** | **Threonine** | **Tryptophan** | **Tyrosine** | **Uracil** | **Urea** | **Valine** |
|  | **MKO/Control** | 0.2952 | 1.0866 | 1.1257 | 1.2065 | 0.8947 | 0.8917 | 1.1486 |
|  | **P-value** | 0.0000 | 0.1078 | 0.1072 | 0.0027 | 0.4797 | 0.4494 | 0.0263 |
